# Metabolome and transcriptome analysis of flavor components and flavonoid biosynthesis in fig female flower tissues (Ficus carica L.) after bagging

**DOI:** 10.1186/s12870-021-03169-1

**Published:** 2021-08-25

**Authors:** Ziran Wang, Miaoyu Song, Zhe Wang, Shangwu Chen, Huiqin Ma

**Affiliations:** 1 College of Horticulture and Landscape, Yunnan Agricultural University, Kunming, 650224 P. R. China; 2 College of Horticulture, China Agricultural University, Beijing, 100193 P. R. China; 3 College of Food Science and Nutritional Engineering, China Agricultural University, Beijing, 100193 P. R. China

**Keywords:** Fig (Ficus carica L.), RNA-seq, Metabolites, Bagging, Female flower tissues, Flavonoids, Flavor substance

## Abstract

**Background:**

Bagging can improve the appearance of fruits and increase the food safety and commodification, it also has effects on intrinsic quality of the fruits, which was commonly reported negative changes. Fig can be regarded as a new model fruit with its relatively small genome size and long fruit season.

**Results:**

In this study, widely targeted metabolomics based on HPLC MS/MS and RNA-seq of the fruit tissue of the ‘Zibao’ fig before and after bagging were analyzed to reveal the metabolites changes of the edible part of figs and the underneath gene expression network changes. A total of 771 metabolites were identified in the metabolome analysis using fig female flower tissue. Of these, 88 metabolites (including one carbohydrate, eight organic acids, seven amino acids, and two vitamins) showed significant differences in fruit tissue before and after bagging. Changes in 16 structural genes, 13 MYB transcription factors, and endogenous hormone (ABA, IAA, and GA) metabolism and signal transduction-related genes in the biosynthesis pathway of flavonoids after bagging were analyzed by transcriptome analysis. KEGG enrichment analysis also determined significant differences in flavonoid biosynthesis pathways in female flower tissue before and after bagging.

**Conclusions:**

This work provided comprehensive information on the composition and abundance of metabolites in the female flower tissue of fig. The results showed that the differences in flavor components of the fruit before and after bagging could be explained by changes in the composition and abundance of carbohydrates, organic acids, amino acids, and phenolic compounds. This study provides new insights into the effects of bagging on changes in the intrinsic and appearance quality of fruits.

**Supplementary Information:**

The online version contains supplementary material available at 10.1186/s12870-021-03169-1.

## Background

Fig (Ficus carica L.) belongs to the Ficus genus of the Moraceae family and is the world’s oldest cultivated fruit tree. It originated in the West Asia area and was introduced to China along the Silk Road thousands of years ago. Fig fruit contains a variety of healthy and beneficial bioactive ingredients, in addition to providing sugar and energy, fig fruit is also rich in dietary fiber and is considered a good source of minerals [1], sterols [2], carotenoids [3], anthocyanins [4], and polyphenols [5]. The development of fig fruit demonstrates a typical double-S-shaped curve, with two rapid growth phases (phases I and III) and one slow growth phase (phase II) between them [6]. Fig is a female flower hermaphrodite plant with diverse fruit color, the edible part is developed from the female flowers of the inflorescence which are covered by the receptacle, botanically, it is a false fruit termed ‘syconium’. The coloration of fig pericarp and female flower tissue shows obvious space-time and expression differences in that the coloration of female flower tissue initiates very early, while the accumulation of anthocyanin in the pericarp is significantly later. The process of anthocyanin accumulation in the pericarp is very fast compared with that of female flower tissue, and the concentration is usually significantly higher. Fruit color is an important index to evaluate the fresh food quality and commodity value of fruits because a bright and attractive appearance is one of the most important factors affecting the choice of growers and consumers [7, 8].

Anthocyanins, water-soluble flavonoids, are important secondary metabolites in plants and have the physiological function of resisting UV damage, pests and diseases, and attracting insect pollination [9]. They also have a strong free radical scavenging effect and have many biological activities, such as cardiovascular protection [10] and anti-tumor properties [11]. Anthocyanins are classified according to the location of phenolic hydroxyl and methyl groups, there are at least 13 types of anthocyanins in nature, six of which, namely petunidin, peonidin, cyanidin, delphinidin, pelargonidin, and malvidin are commonly found. Currently, these six types of anthocyanins and their derivatives are about 95% of the total amount of anthocyanins in nature. The biosynthesis pathway of anthocyanins has been studied in horticultural plants, such as apple, grape and tomato in detail [12]. These studies have identified important upstream structural genes, such as chalcone synthase (CHS) and chalcone isomerase (CHI), and downstream structural genes, such as dihydroflavonol reductase (DFR), anthocyanidin synthase (ANS), and flavonoid 3-O-glycosyl transferase (UFGT), in the flavonoid biosynthesis pathway. The expression of anthocyanin synthetic structural genes is regulated by a variety of transcription factors (TFs). According to the conserved domains contained in TFs, they are roughly divided into MYB, bHLH, WD40, MADS-box, and bZIP families [13], among which the most studied is the MYB family. MYB is one of the most characteristic families of TFs, widely distributed in all eukaryotes, including animals, plants, and fungi. MYB proteins can be divided into four types based on the number of MYB repeats:1 R-MYB, R2R3-MYB, R1R2R3-MYB, and 4 R-MYB [14]. The studies on the regulation of anthocyanin biosynthesis in the MYB TF family mainly focus on the R2R3-MYB TF, and the study on the regulation of anthocyanin in fruit trees by the R2R3-MYB TF began late. In 2006, Espley et al. found a light-induced anthocyanin synthesis gene *MdMYB1* in apple, followed by two types of genes regulating anthocyanin biosynthesis in pulp, *MdMYB10* [15] and *MdMYB110a* [16]. With the separation and functional validation of the key TFs, *MdMYB1* and *MdMYB10*, of anthocyanin in apple, the homologous genes were isolated successively in plants such as strawberry [17], peach [18], pear [19], sweet cherry [20], and pomegranate [21]. R2R3-MYB regulation can be divided into activation and inhibition, for example, *FaMYB1* in strawberries [22], *AtMYBL2* in *Arabidopsis* [23], and *VvMYBC* in grapes [24], which inhibit anthocyanin synthesis.

Bagging is used frequently in the cultivation and management of fruit trees and is widely applied in apple, pear, grape, and other fruit trees. Fruit bagging can improve the appearance and intrinsic quality of fruit to a certain extent, reduce pesticide residues, and improve the food safety and commodification of fruit. When there is a difference in light sensitivity between the pericarp and pulp coloration, bagging can change the influence of external environmental factors, especially the light signal, on anthocyanin synthesis. Bagging also has effects on intrinsic quality of the fruits, which was commonly reported negative changes. Fig can be regarded as a new model fruit with its relatively small genome size and long fruit season. As a non-photosensitive part, the anthocyanin synthesis regulation gene and the appearance of flavor components of the female flower tissue of fig may have another regulatory mechanism. *Arabidopsis* with mutant *cop1* can maintain anthocyanin synthesis under dark conditions, PAP1 and PAP2 activate structural genes PALs, CHS, CHI, F3H, F3′H, ANS, and DFR, promoting anthocyanin biosynthesis [25]. Bagging has no great influence on anthocyanin accumulation in non-photosensitive fruits, but it has a remarkable impact on the formation of flavor components and taste. The appearance of the fruit after bagging improves significantly, however, the taste and flavor of the fruit are another important factors affecting customer choice. Fruit odor (caused by volatile compounds such as phenols and alcohols) and taste (determined by the proportion and concentration of sugars, organic acids, and amino acids) contribute to the formation of fruit flavor [26]. Previous studies have focused on the identification of color, soluble solids, total sugar, and total acid components of the fruit after bagging [27, 28], however, research on the changes of internal transcription and metabolite networks in the fruit after bagging is lacking.

In this study, the fig cultivar ‘Zibao’ with both colored pericarp and pulp was used as the research material. To better understand the effect of bagging treatment on fruit color quality and flavor components, the transcriptome and widely targeted metabolome were used jointly to analyze bagged fruit. The changes of related secondary metabolites including carbohydrates, organic acids, and amino acids in female flower tissues before and after bagging in fig fruit were identified by the metabolome, and the differential changes of related MYB TFs, structural genes and endogenous hormone (ABA, IAA, and GA) metabolism and signal transduction-related genes in the anthocyanin synthesis pathway were analyzed through the transcriptome. This study investigated how bagging affected the molecular mechanism involved in the color quality and the formation of flavor components in fig fruit, which provides a theoretical basis for in-depth analysis into the gene regulation mechanism of fig fruit coloration and further delivers a reference to produce high-quality fig fruit.

## Results

### Widely targeted metabolome and KEGG classification analysis of differential metabolites

**Fig. 1:**
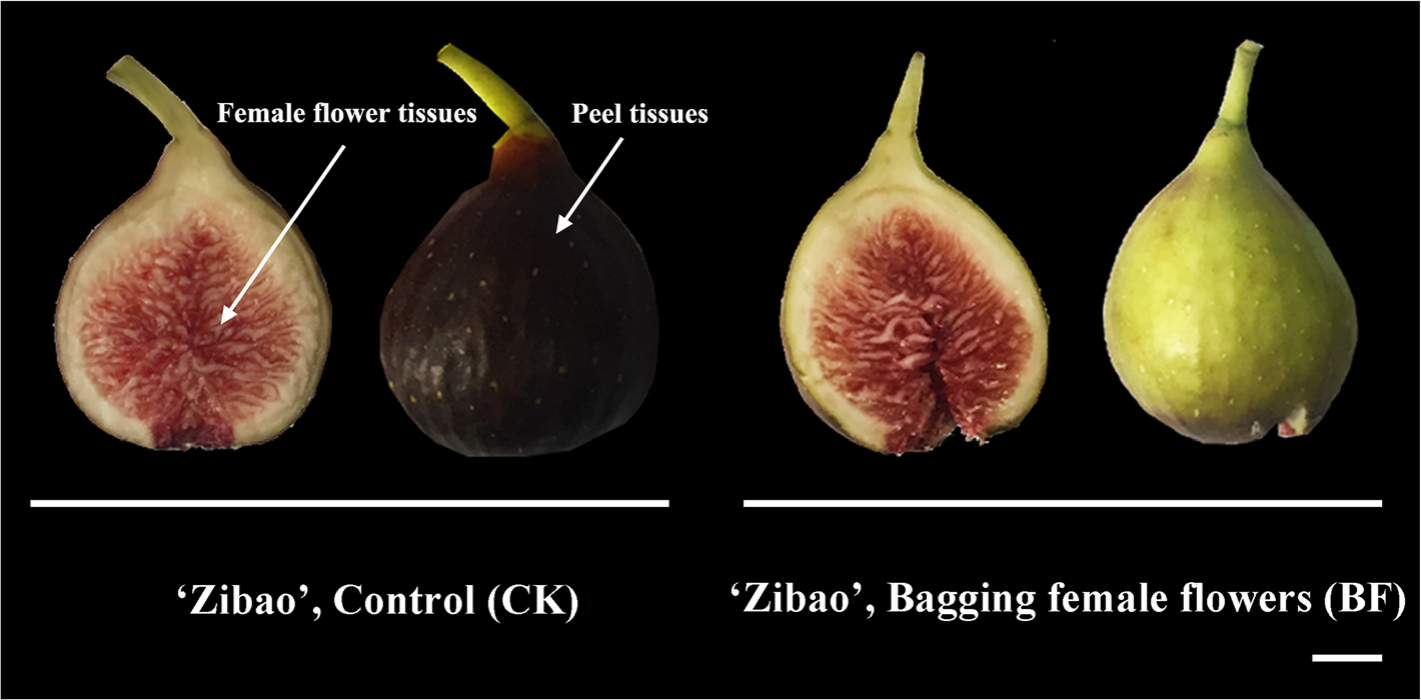
The mature female flower tissues from ‘Zibao’ (control, CK) cultivars and the after bagging female flower tissues (BF). Scale bar = 1 cm

**Fig. 2:**
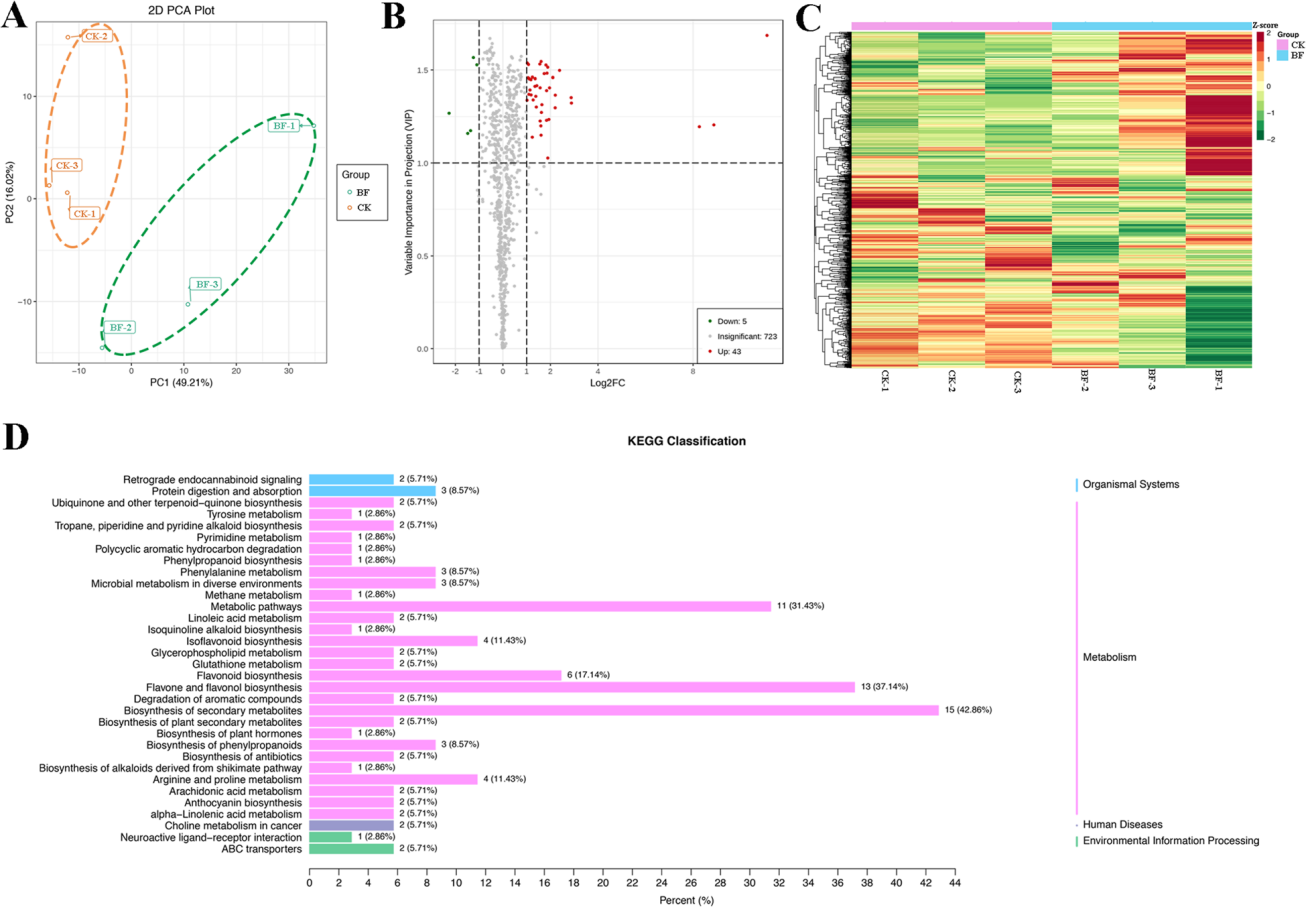
Preliminary analysis of metabolomics data. (A) PCA score plot metabolite profiles from ‘Zibao’ Fig. (CK) and the after bagging female flower tissues (BF). (B) Volcano plots of SCMs between CK and BF. (C) Cluster analysis of metabolites from samples of CK and BF. The colour indicates the level of accumulation of each metabolite, from low (green) to high (red). The Z-score represents the deviation from the mean by standard deviation units. (D) KEGG classification of DEGs between CK and BF

The coloration of mature fig pericarp must be illuminated to promote anthocyanin accumulation under pulsed light irradiation [29]. The subsequent experiments showed that the coloration of fruit pericarp was regulated by the light signal, and the accumulation of anthocyanin stopped almost completely under bagging conditions. However, the coloration of the female flower tissue of fig was not affected by the light signal, and the development of anthocyanin in the female flower was barely affected under the condition of fruit bagging, and the content of anthocyanins of female flower in the mature stage was basically the same as that of the control group, for the red cultivar; the female flower tissue in the mature stage kept bright red (Fig. 1). Although there is no significant difference in the color of female flower tissue, the flavor and taste are still slightly different. To explore the effect of bagging on fruit flavor components, the secondary metabolites of samples before and after bagging were analyzed by a widely targeted metabolome. A total of 771 compounds in 16 classes were detected (Table S2). Principal component analysis (PCA) was conducted with 771 metabolites, 49.21% of PC1 and 16.02% of PC2. PCA separated the two group of female flower samples, the significance was 0.01 (Fig. 2A). With Log2FC ≥ 2 and ≤ 0.5, and VIP ≥ 1, 43 upregulated and five downregulated metabolites were identified (Fig. 2B). Using metabolite concentration data for the cluster analysis of a stratified heat map of the samples, it was observed that all biological replicates were grouped together (top of the figure), which indicated a high reliability of the resulting metabolome data (Fig. 2C). Interestingly, a clear distinction between the unbagged fruit samples (CK) and the bagged fruit samples (BF) were observed, suggesting a distinct difference in metabolite characteristics in the two samples. The metabolites (left side of the figure) were also clustered into two main groups, showing opposite accumulation levels between red female flower tissue samples before and after bagging. A total of 771 metabolites were mapped to the KEGG database and the results indicated that most metabolites were associated with “metabolism”. Some metabolites were classified as “biological systems” or “human diseases”, which suggested that some metabolites in the female flower tissues of fig may have potential health effects. KEGG enrichment analysis showed that there were mainly 35 groups, among which, the metabolites of “biosynthesis of plant secondary metabolites”, “flavone and flavonol biosynthesis”, and “metabolic pathway” were three groups with significant difference of female flower tissues before and after bagging (*p* < 0.05) (Fig. 2D).

### Phenylpropanoids, flavone, flavonol, flavanone, and anthocyanins

A total of 234 substances were detected by flavonoid metabolite analysis, of which 32 reached a level of significant difference. For further mining differential metabolites, data was analyzed by PLS-DA (Fig. S1). The PLS-DA score map exhibited a distinct separation between groups and obvious clustering within the group, which further suggested that the difference between the two groups was significant. The quality parameters of the model with two principal components were as follows: R2X = 0.643, R2Y R2X = 0.995, and Q2R2X = 0.739, which indicated that the current model had a better ability to interpret and predict data. Using VIP ≥ 1.0 and | Log_2_FC | ≥ 1 as a threshold for significant differences, 32 flavonoid metabolites were identified from ‘CK vs. BF’ samples as significantly differentially accumulated, including twelve flavones, nine phenylpropanoids, five flavonols, three anthocyanins, and one flavanone (Table 1). The cyanidin O-acetylhexoside and cyanidin 3-O-malonylhexoside were found increased by 1.13 times and 1.06 times compared with CK in the female flower tissue of the bagged fruit, which explained the slightly darker color of the bagged fruit compared with the CK. The phenylpropanoid biosynthesis pathway is upstream of the anthocyanin and flavonoid biosynthesis pathway. Nine phenylpropanoid secondary metabolites were identified, among which the expression of six increased and three decreased. The phenethyl caffeate, angelicin, and methyl p-coumarate contents in female flower tissues increased 8.26 times, 2.87 times, and 2.20 times in ‘CK vs. BF’, while the contents of 3,4,5- trimethoxycinnamic acid, ioacteoside, and 3,4-dihydrocoumarin decreased 2.26,1.48, and 1.09 times, respectively. Twelve flavonoids were detected in female flower tissue, 11 of which increased. The biggest differences were shown in three groups: apigenin, nobiletin, and tangeretin. Among them, the upstream substrate apigenin of luteolin increased significantly in BF by 11.11 times.

**Table 1:** Differentially accumulated phenolic compounds with VIP (variable importance in projection) ≥ 1, and | Log_2_FC| ≥ 1 as for upregulation/downregulation in the ‘Zibao’ female flower tissues (CK) by bagging (BF)

Component name	Metabolite name	Content		Log_**2**_FC (BF vs. CK)	VIP	Type
		CK	BF			
**Anthocyanin**	Cyanidin O-acetylhexoside	1.91E+ 05	4.13E+ 05	1.11E+ 00	1.45E+ 00	up
	Cyanidin 3-O-malonylhexoside	2.91E+ 05	6.08E+ 05	1.06E+ 00	1.46E+ 00	up
**Phenylpropanoids**	Phenethyl caffeate	9.00E+ 00	2.77E+ 03	8.26E+ 00	1.20E+ 00	up
	Angelicin	1.84E+ 03	1.35E+ 04	2.87E+ 00	1.35E+ 00	up
	Methyl p-coumarate	2.29E+ 03	1.05E+ 04	2.20E+ 00	1.30E+ 00	up
	Cinnamic acid	4.31E+ 04	1.54E+ 05	1.84E+ 00	1.23E+ 00	up
	Psoralen	5.76E+ 03	1.76E+ 04	1.61E+ 00	1.55E+ 00	up
	trans-Cinnamate	5.38E+ 04	1.63E+ 05	1.60E+ 00	1.20E+ 00	up
	3,4-Dihydrocoumarin	4.61E+ 04	2.16E+ 04	-1.09E+ 00	1.53E+ 00	down
	Isoacteoside	8.83E+ 03	3.17E+ 03	-1.48E+ 00	1.16E+ 00	down
	3,4,5-Trimethoxycinnamic acid	2.09E+ 04	4.35E+ 03	−2.26E+ 00	1.27E+ 00	down
**Flavone**	Apigenin	9.00E+ 00	1.99E+ 04	1.11E+ 01	1.69E+ 00	up
	Nobiletin	8.70E+ 03	3.11E+ 04	1.84E+ 00	1.39E+ 00	up
	Tangeretin	5.89E+ 03	2.07E+ 04	1.81E+ 00	1.53E+ 00	up
	Apigenin 7-O-glucoside (Cosmosiin)	1.17E+ 05	3.43E+ 05	1.56E+ 00	1.53E+ 00	up
	Luteolin 7-O-glucoside (Cynaroside)	3.35E+ 05	9.00E+ 05	1.43E+ 00	1.42E+ 00	up
	Velutin	1.45E+ 04	3.78E+ 04	1.38E+ 00	1.36E+ 00	up
	Chrysin O-hexoside	1.66E+ 04	4.24E+ 04	1.36E+ 00	1.45E+ 00	up
	Chrysin 5-O-glucoside (Toringin)	1.68E+ 04	4.06E+ 04	1.27E+ 00	1.34E+ 00	up
	Acacetin	6.46E+ 04	1.38E+ 05	1.09E+ 00	1.37E+ 00	up
	Chrysoeriol 5-O-hexoside	1.71E+ 05	3.60E+ 05	1.08E+ 00	1.53E+ 00	up
	Luteolin	4.97E+ 03	1.02E+ 04	1.03E+ 00	1.54E+ 00	up
	Tricin 5-O-rutinoside	8.81E+ 03	3.44E+ 03	−1.36E+ 00	1.17E+ 00	down
**Flavonol**	Kaempferol 3-O-rhamnoside (Kaempferin)	1.20E+ 04	4.00E+ 04	1.74E+ 00	1.48E+ 00	up
	Kaempferol 7-O-rhamnoside	1.78E+ 04	5.28E+ 04	1.57E+ 00	1.41E+ 00	up
	Kumatakenin	2.33E+ 04	5.93E+ 04	1.35E+ 00	1.41E+ 00	up
	Morin	3.29E+ 05	7.77E+ 05	1.24E+ 00	1.14E+ 00	up
	Kaempferol 3-O-galactoside (Trifolin)	2.10E+ 05	4.75E+ 05	1.18E+ 00	1.45E+ 00	up
**Flavanone**	Pinocembrin (Dihydrochrysin)	2.19E+ 04	1.62E+ 05	2.88E+ 00	1.32E+ 00	up
**Isoflavone**	2′-Hydroxygenistein	3.50E+ 03	1.30E+ 04	1.89E+ 00	1.51E+ 00	up
	Sissotrin	1.64E+ 04	7.01E+ 04	2.10E+ 00	1.46E+ 00	up
	Prunetin	6.50E+ 04	1.43E+ 05	1.14E+ 00	1.39E+ 00	up

### Carbohydrates, amino acids, organic acids, and vitamins

Flavor and nutrient components are mainly composed of carbohydrates, organic acids, and amino acids. VIP ≥ 1, and fold change ≥1.6 and ≤ 0.6 was set as the threshold of significant difference, a total of 19 significantly differentially accumulated flavor-related metabolites were identified from the ‘CK vs. BF’ samples, including one carbohydrate, seven amino acids and derivatives, eight organic acids and derivatives, and three vitamins and derivatives (Table 2). D(−)-Threose increased 3.70 times more than that of CK in the female flower tissues of bagged fruit. In addition, the overall carbohydrate accumulation in fig female flower tissues was found decreased after bagging treatment (1 increment, 6 reduction), with D-Glucose 6-phosphate and D-Fructose 6-phosphate as important carbohydrates responsible for the flavor composition in female flower tissues (Table S3). Seven substances of amino acids and derivatives were screened from the female flower tissue before and after bagging, two of which increased, while five decreased. In the significantly decreased metabolites, L-aspartic acid, CYS-GLY, L-homocysteine, phenylacetyl-L-glutamine, and aspartic acid all reached more than five times the significant difference level, which indicated that the bagged fruit had less sugar and acid comparing with the CK, leading to a reduction of flavor. Among them, the upstream substrate apigenin of luteolin increased significantly in BF by 11.11 times. The phenylpropanoid biosynthesis pathway is upstream of the anthocyanin and flavonoid biosynthesis pathway. In ‘CK vs. BF’, eight significantly different organic acids and derivatives were screened, of which seven were increased and one was decreased. Among these, the α-hydroxyisobutyric acid, (S)-(−)-2-hydroxyisocaproic acid, and isochlorogenic acid B contents increased 4.61 times, 3.05 times, and 2.28 times, respectively, while the phosphoenolpyruvate trisodium salt content decreased 1.46 times.

**Table 2:** Differentially accumulated carbohydrates, amino acids, organic acids and vitamins compounds with VIP (variable importance in projection) ≥ 1, and fold change ≥1.6 and ≤ 0.6 as for as for upregulation/downregulation in the cv. ‘Zibao’ CK and BF

Component name	Metabolite name	Average content		Fold change (BF vs. CK)	VIP	Type
		CK	BF			
**Carbohydrates**	D(−)-Threose	5.60E+ 03	8.46E+ 03	3.70E+ 00	1.03E+ 00	up
**Amino acid and derivatives**	L-Tyramine	2.01E+ 05	7.84E+ 05	3.90E+ 00	1.40E+ 00	up
	N-γ-Acetyl-N-2-Formyl-5-methoxykynurenamine	2.63E+ 05	4.23E+ 05	1.61E+ 00	1.47E+ 00	up
	L-Aspartic acid	1.19E+ 07	7.10E+ 06	5.98E-01	1.07E+ 00	down
	CYS-GLY	5.61E+ 04	3.25E+ 04	5.79E-01	1.49E+ 00	down
	L-Homocystine	2.09E+ 05	1.19E+ 05	5.72E-01	1.28E+ 00	down
	Phenylacetyl-L-glutamine	1.61E+ 05	9.01E+ 04	5.61E-01	1.06E+ 00	down
	Aspartic acid	1.05E+ 07	5.83E+ 06	5.55E-01	1.15E+ 00	down
**Organic acids and derivatives**	α-Hydroxyisobutyric acid	1.80E+ 05	8.28E+ 05	4.61E+ 00	1.37E+ 00	up
	(S)-(−)-2-Hydroxyisocaproic acid	2.59E+ 04	7.88E+ 04	3.05E+ 00	1.15E+ 00	up
	Isochlorogenic acid B	7.36E+ 04	1.68E+ 05	2.28E+ 00	1.37E+ 00	up
	Chlorogenic acid methyl ester	2.26E+ 04	4.39E+ 04	1.94E+ 00	1.14E+ 00	up
	2,3-Dihydroxybenzoic acid	9.30E+ 05	1.74E+ 06	1.87E+ 00	1.41E+ 00	up
	2-Hydroxybutanoic acid	3.35E+ 04	5.67E+ 04	1.69E+ 00	1.48E+ 00	up
	D-Pantothenic dcid	1.39E+ 04	2.35E+ 04	1.69E+ 00	1.19E+ 00	up
	Phosphoenolpyruvate trisodium salt	7.34E+ 04	4.08E+ 04	5.56E-01	1.46E+ 00	down
**Vitamins and derivatives**	delta-Tocopherol	2.68E+ 03	9.71E+ 03	3.62E+ 00	1.48E+ 00	up
	Thiamine pyrophosphate	8.41E+ 03	1.44E+ 04	1.71E+ 00	1.46E+ 00	up
	Orotic acid	1.52E+ 05	2.59E+ 05	1.71E+ 00	1.57E+ 00	up

### RNA-seq analysis

The control and treatment group of ‘Zibao’ fig female flower tissue in a total of two samples, each with three biological replicates, were sequenced in this study. The control group (CK) was the female flower tissue at the end of phase III, and the treatment group was the female flower tissue after bagging and named bagged flower (BF). The double-terminal reading of the cDNA library of the two samples was performed using the Illumina HiSeq 4000 platform, which produced 7,394,743,914 and 9,582,970,682 paired-end original reads of 200 bp in length. The low-quality reads were deleted and the joint sequences were removed, resulting in the acquisition of 6,747,389,564 and 9,137,841,606 clean data for CK and BF libraries, respectively. Mapping ratios compared to reference databases were 91.37 and 91.32%, respectively (Table S4). Different genes were identified between the two groups and filtrated and corrected by FDR < 0.05 and | log2FC | ≥ 2. By comparing the number of different genes between the control and bagged group between the pericarp and the female flower, 2389 differentially expressed genes were found in the ‘CK vs. BF’, among which the number of up-regulated genes was slightly higher than that of down-regulated genes, 1208 and 1181, respectively (Fig. S2 A). Gene Ontology (GO, http://www.geneontology.org/) annotation of the DEGs found that 1154 unigenes were annotated to ‘Biological Process’, 674 unigenes were annotated to ‘Cellular Component’, and 572 unigenes were annotated to ‘Molecular Function’ (Fig. S2 B). To identify the biological pathways activated in fig female flowers, the normative reference pathway for annotation sequences to the KEGG database was chosen. In KEGG pathways, protein processing in the endoplasmic reticulum, plant hormone signal transduction, and plant-pathogen interaction pathways were significantly changed in the ‘CK vs. BF’ group with corrected *P*-value ≤0.05 (Table S5, Fig. S2 C).

### Flavonoid biosynthesis pathway and transcriptional regulation

**Fig. 3:**
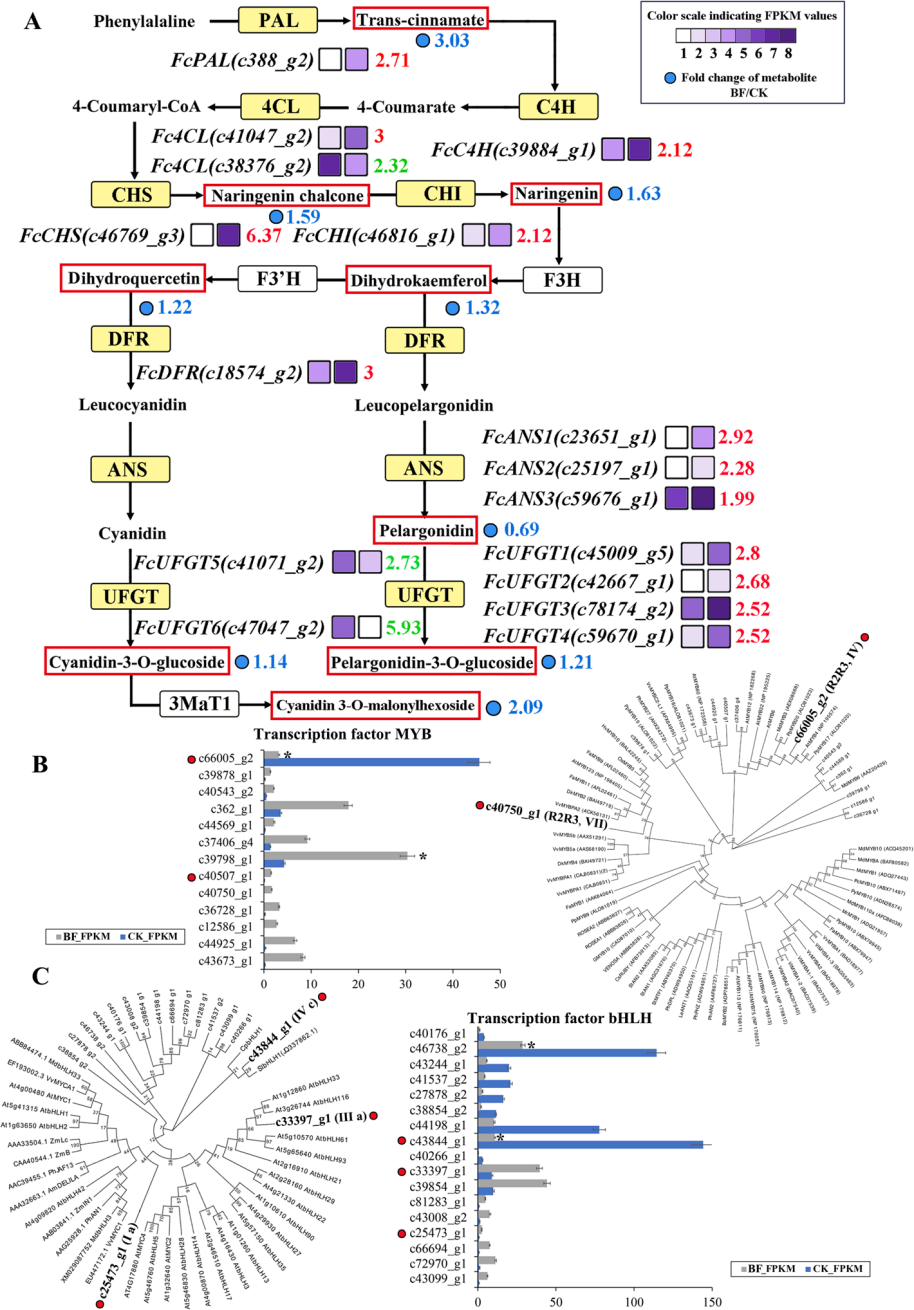
Transcript profiling FPKM and phylogenetic tree of structural genes and transcription factors in the flavonoid biosynthesis pathway in cv. ‘Zibao’ (CK) and the after bagging female flower tissues (BF). (A) Reconstruction of the phenylpropanoid-flavonoid biosynthetic pathway with the significantly differentially expressed structural genes (yellow). Enzyme names, log2 (expression ratio) and expression patterns are indicated at the side of each step. The expression pattern for each uni-transcript is shown on two grids, with the left one representing the FPKM value of ‘CK’, and the right one of ‘BF’. Grids with 8 different gray-scale levels show the FPKM value, with the RPKM values 0–10, 10–20, 20–40, 40–80, 80–160, 160–320, 320–640 and 640–1280 represented by gray-scale levels 1–8, respectively. (B) The expression patterns of the candidate MYB transcription factor involved in the regulation of flavonoid compounds in CK vs. BF and phylogenetic clustering of the recruited R2R3-MYBs with anthocyanin biosynthesis-related MYBs from other plants. (C) The expression patterns of the candidate bHLH transcription factor involved in the regulation of flavonoid compounds in CK vs. BF and Phylogenetic clustering of the recruited bHLHs with anthocyanin biosynthesis-related bHLHs from other plants. *P < 0.05

The expression of nine structural genes of the flavonoid biosynthesis pathway (*PAL*, *C4H*, *4CL*, *CHS*, *CHI*, *F3H*, *F3′H*, *DFR*, *ANS*, and *UFGT*) play a critical role in anthocyanin biosynthesis. To better understand the time-space difference of coloration between the pericarp and the female flower of fig, 16 important structural genes differentially expressed in the female flower tissues of the fig ‘Zibao’ were selected. Of them, PAL, *FcPAL* (c388_g2), was significantly increased 2.71 times after bagging, C4H, *FcC4H* (c39884_g1), was significantly increased 2.12 times after bagging, and CHS, *FcCHS* (c46769_g3), was significantly upregulated 6.37 times in female flower tissue after bagging. *FcCHI* (c46816_g1) was downregulated 2.12 times in female flower tissue. No significant changes were found in F3H and F3′H expression in female flower tissues after bagging. In ‘CK vs. BF’, *FcDFR* (c18574_g2) was significantly upregulated by three times. Three *ANS* genes were selected, in ‘CK vs. BF’, *FcANS1*, *FcANS2*, and *FcANS3* all showed a trend of upregulation, with 2.92 times, 2.28 times, and 1.99 times, respectively. There were six *UFGT* genes, four (*FcUFGT1, FcUFGT2, FcUFGT3*, and *FcUFGT4*) were significantly upregulated 2.8 times, 2.68, 2.52, and 2.52 times, respectively in ‘CK vs. BF’, two *UFGT* genes, *FcUFGT5* (c41071_g2) and *FcUFGT6* (c47047_g2), were significantly downregulated 2.73 times and 5.93 times, respectively in ‘CK vs. BF’ (Fig. 3A). Anthocyanidin 3-O-glucosyltransferase catalyzes cyanidin to cyanidin-3-O-glucoside (5.93-fold downregulation). Cyanidin-3,5-O-diglucoside can be glycosylated from cyanidin-3-O-glucoside (2.09-fold downregulation) or cyanidin-5-O-glucoside; UDP-glycosyltransferase 75D1 catalyzes pelargonidin to pelargonidin-3-O-glucoside (1.21-fold downregulation). In addition, increased content of the phenylpropanoids and flavone, flavonol and isoflavone were measured in the fig female flower tissue (Table 1, Fig. 3A).

Gene expression at the transcriptional level plays an important role in regulating and controlling many biological processes, TFs are the key to the regulation of secondary metabolite genes. After bagging, the original red pulp of the fig ‘Zibao’ did not change, showing non-light dependency. In ‘CK vs. BF’, 13 MYB and 17 bHLH family members showed significant differences. MYB TFs are widely found in plants and are involved in almost all aspects of plant development and metabolism. In these MYB families, the expression of 12 genes was upregulated, of which c43673_g1, c12586_g1, c44925_g1, and c36728_g1 were different by 4.87, 4.81, 4.24, and 4.14 times, respectively, while c66005_g2 was downregulated 3.8 times (Fig. 3B). The highly expressed MYB c66005_g2 in CK was closely related to the flavonoid MYB repressor PpMYB20 (Fig. 3B), whereas c40750_g1 clustered with the anthocyanin activator groups, with high similarity to VvMYB5a, VvMYB5b and VvMYBPA2, which regulate anthocyanin biosynthesis in grape [30].

The basic helix-loop-helix (bHLH) family is the second largest family of TFs in plants and has many functions, including regulating flower organ development, photomorphogenesis, epidermal hair, and stomatal formation, plant hormone response, and flavonoid metabolism [13] Eight genes from the bHLH family were significantly upregulated in the ‘CK vs. BF’, c66694_g1(4.94), c81283_g1(3.68), c72970_g1(3.66), c43099_g1(3.42), c25473_g1(2.92), c43008_g2(2.72), c39854_g1(2.16), and c33397_g1(2.12), while nine genes showed a trend of significant downregulation with c43844_g1 and c40266_g1 decreased 3.71 and 4.39 times, respectively (Fig. 3C). By constructing a phylogenetic evolutionary tree, the high expression of bHLH c43844_g1 in CK was closely associated with the carotenoid biosynthesis activator CpbHLH1 in papaya (Fig. 3C) [31]. In addition, c25473_g1 clustered with VvMYC1, MdbHLH3 and MdbHLH33. The co-expression of bHLH transcription factor VvMYC1 and VvMYBA1 can accumulate anthocyanins in grape suspension cells [32]. The interaction of MdbHLH3 and MdbHLH33 with MYB TFs is involved in the regulation of anthocyanin synthesis in apple fruit [33]. The bHLH family members are associated with anthocyanin biosynthesis in fruit trees and have been shown to interact with MYB TFs to regulate fruit color.

### Changes of endogenous plant hormone metabolism and signal transduction genes after bagging

**Fig. 4:**
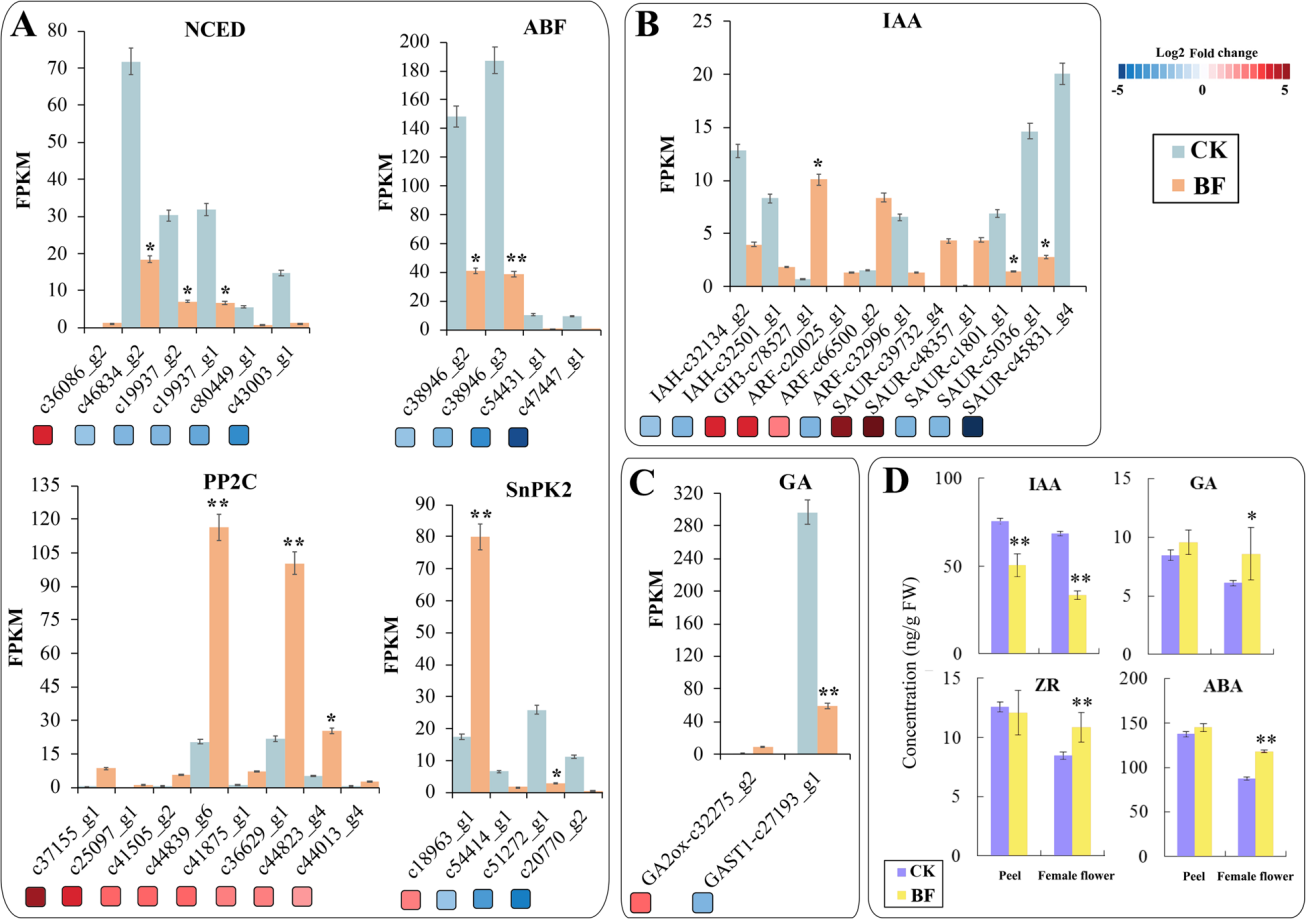
Expression profiles of the differentially expressed endogenous hormone metabolism and signal transduction-related genes in cv. ‘Zibao’ (CK) and the after bagging female flower tissues (BF). (A) Comparison of ABA metabolism and signal transduction-related differentially expressed genes (DEGs) found in CK vs. BF. (B) Comparison of IAA metabolism and signal transduction-related DEGs found in CK vs. BF. (C) Comparison of GA metabolism and signal transduction-related DEGs found in CK vs. BF. Blue and red boxes indicate downregulated and upregulated transcripts, respectively. (D) Phytohormone content of fig peel and female flower by ELISA. **P < 0.01; *P < 0.05. FW, fresh weight

The endogenous hormones GA and ABA are transformed from glucose molecules, a direct product of photosynthesis, through plant isoprene biosynthesis, which regulates anthocyanin biosynthesis [34]. In ABA biosynthesis, except for c36086_g2, which was significantly upregulated, all of the other four 9-cis-epoxycarotene dioxygenase (NCED) genes were significantly downregulated. Eight genes were annotated as possible protein phosphatase 2 C (PP2C), significantly upregulated. Two genes (c2285_g1 and c25449_g1) were annotated as ABA-activated protein kinase 2(SNRK2) in female flowers, and four ABRE binding factor (ABF) genes were significantly downregulated after bagging. One ABA 8′- hydroxylase (ABA 8′-h) gene (c23609_g1) was significantly downregulated 3.17 times for ABA catabolism (Fig. 4A). In IAA biosynthetic genes, three were annotated as indole-3-acetic acid-inducible protein (ARG7) genes: one significantly upregulated gene c39732_g4, two significantly downregulated genes c1801_g1 and c45831_g4, in three auxin response factors (ARF), c20025_g1 and c20025_g2 were upregulated, and c32996_g1 was downregulated. Moreover, there was one upregulated Gretchen Hagen3 (GH3) gene c78527_g1, one upregulated signal transduction auxin-instream vector (AUX1) gene, and two IAA-amino acid hydrolase (IAH) genes c32134_g2 and c32501_g1 (Fig. 4B). Gibberellin (GA) plays an important role in the ripening of strawberries and sweet cherries [35]. The GA degradation gene GA2 oxidase (GA2ox) c32275_g2 was significantly up-regulated by 2.88 times in the ‘CK vs. BF’, and the GA stimulus transcript (GASTI) gene c27193_1 was inversely regulated, which decreased 2.32 times in the bagged female flower tissue (Fig. 4C). The level of four phytohormones in CK and BF pericarp and female flower tissue was determined by enzyme-linked immunoassay (ELISA). The level of active IAA was higher in CK than in BF in female flower tissues and pericarp, and the difference reached a highly significant level (*P* < 0.01). The GA content measured in this experiment was the sum of GA_1_ and GA_3_. In BF vs. CK, GA level in the pericarp was significantly higher than that in the female floral tissue, after bagging, GA level in the pericarp and female floral tissue increased moderately. The ZR content in the pericarp and female flower tissues showed opposite trends, decreased after bagging in the peel and slightly increased in the female flower tissues, and the difference was significant (*P* < 0.01) (Fig. 4D).

### The qRT-PCR validation

To verify the key results of the RNA-seq, 15 genes from flavonoid biosynthesis pathways and endogenous hormone signal transduction pathway were selected for validation, their expression levels in CK and BF were analyzed by qRT-PCR (Fig. S3). The results confirmed that the expression level of the genes was similar to that of the RNA-seq results, and there was good agreement with the up- and downregulated gene expression trend revealed by RNA-seq.

## Discussion

Bagging can improve the sensitivity of the fruit to light and affect the intrinsic and appearance quality of the fruit while changing the microenvironment of fruit development. The flavor components of fruit are generally related to the contents of sugar, organic acids, and phenols. Previous studies have focused on the effect of bagging on pericarp color, while studies on flavor changes have just focused on several specific types of metabolites, such as sugars (fructose, glucose, and sucrose), organic acids, amino acids, and alcohols [36, 37]. To date, the overall variation of secondary metabolites of fig fruits by bagging has not been studied. In this study, an LC-MS/MS-based widely targeted metabolomics approach was used to understand the flavor changes of fig fruit after bagging. A total of 771 metabolites were identified, 88 of which were accumulated in female flower tissues after bagging in a differentially expressed manner. A large amount of carbohydrates were confirmed in the tissues of female fig flowers (Table S2), including 20 sugars, 12 of which had significantly reduced concentrations in female flower tissue after bagging, including D-glucose 6-, D-fructose 6-, ribulose-5-phosphate, glucose− 1-phosphate, and D-fructose 6-phosphate-disodium salt. These five reduced sugar types are the main sugar types that form the flavor of fig fruit, while 115 types of organic acids were identified, the concentration of half (52) were significantly reduced in female flower tissue after bagging, which can partly explain the change in the flavor of the fruit. The composition and richness of amino acids are the key indexes of nutritional quality and are also important for determining flavor [38]. Previous studies have identified 12 amino acids in fig fruit [39]. However, 99 amino acids were identified by widely targeted metabolome analysis, seven of these accumulated differences between CK and BF (Table 2). Five of these amino acids (L-aspartic acid, CYS-GLY, L-homocysteine, phenylacetyl-L-glutamine, and aspartic acid) were significantly reduced after bagging (Table 2). Therefore, the results show that differences in amino acid composition and abundance can also lead to changes in flavor matter. Transcriptome analysis revealed seven differentially expressed genes related to amino acid synthesis and transporter, three of which were up-regulated and four were down-regulated. Specifically, amino acid transporter YPQ gene was down-regulated 2.93-fold (Table S6).

Plant hormones play important roles in inducing anthocyanin accumulation. Plant hormones usually interact with light signaling pathways to regulate plant growth. E3 ubiquitin ligase-based constitutively photomorphogrnic1 (COP1) is a decisive inhibitor of photomorphogenesis in the process of signal transduction, and a ‘molecular switch’ located downstream of the photoreceptor. Under dark conditions, COP1 proteins enter the nucleus and can form complexes with suppressor of phyA-105 (SPA1) [40]. Then, ubiquitin degrading photomorphogenesis promotes TF HY5, and inactivates them or leaves their areas of action, thereby regulating anthocyanin synthesis [41]. Under light condition conditions, COP1 transfers from the nucleus to the cytoplasm and HY5 accumulation initiates photomorphogenesis. ABA metabolism and signal transduction gene synthesis were significantly differentially expressed by light regulation in this study. Studies have demonstrated that members of the ABA pathway can be associated with both HY5 and PIF3, indicating that ABA may be the most important plant hormone in light-dependent anthocyanin biosynthesis [35]. The GO enrichment analysis of the DEGs described above indicates that, functionally, GA participates in photo-mediated anthocyanin biosynthesis (Fig. 4). Meanwhile, HY5 and PP2CA, as well as PIF3 and PYL10, are co-expressed with other TFs and structural genes involved in anthocyanin biosynthesis. Similarly, members of possible interaction pathways HY5-SLY1-GASA3 and PIF3-GID1A are co-expressed with anthocyanin biosynthetic genes. At present, it has been found that ABA plays an important role in the development, maturation, and postharvest stage of fig female flower tissue. ABA is produced rapidly in female flower tissue in figs before color conversion and reaches the highest level before the commercial maturation period, whereas the anthocyanin content changes later than ABA. The ABA synthesis inhibitor NDGA inhibits the accumulation of anthocyanin, which indicates that ABA is one of the important factors to promote fruit coloring during the normal development of fig [35].. Hence, this study proposes a co-expression network that involves ABA-HY5-MYB in the biosynthesis of anthocyanin in female flower tissue. To understand whether the change of the expression level of numerous hormone metabolism and synthesis-related genes in fruit affects the change of flavor components in a later stage of fruit, future studies will be carried out on hormone metabolites in fruit after bagging. This study will lay the foundation for exploring the molecular and metabolic mechanisms of plant hormones and fruit flavor component formation.

## Conclusions

In this study, HPLC-MS/MS-based metabolome and transcriptome analysis were successfully performed to systematically compare flavor component differences and flavonoid biosynthesis changes before and after bagging in fig fruit. This work provided comprehensive information on the composition and abundance of metabolites in the female flower tissue of fig. It also preliminarily explored the difference in patterns of the space-time coloration of different fig tissues. To identify the various regulatory patterns of structural genes and MYB TFs involved in flavonoid biosynthesis pathways, as well as component differences and concentration changes of carbohydrates, organic acids, amino acids, phenols, and alcohols in female flower tissue, the underlying causes of changes in fruit flavor quality after bagging were determined.

## Methods

### Plant material and treatment

The common fig cultivar ‘Zibao’ (the formal identification was approved by the State Forestry Administration of China, the new variety right number [20150145]) was planted at the Shangzhuang Experimental Station (40°23′ N, 116°49′ W) of China Agricultural University, Haidian District, Beijing, China. The original source of the fig materials used from Weihai Changshoukang Food Co., Ltd. in Shandong province. Experimental research on fig comply with China and Shandong province local legislation. The trees have been cultivated for 5 years and planted in a greenhouse with a row spacing and plant spacing of 2 m × 3 m. Double-layer opaque paper bags with a black inside and light brown outside (150 mm × 150 mm, Zhengguo Paper Bag, Zhengzhou Fruit Research Institute, Chinese Academy of Agricultural Sciences) were used for shading the figs. Figs were sampled in the late stage of phase III and termed CK (natural growth of female flowers) and BF (bagged female flowers). There were three biological replicates per sample each with 30 fruits collected randomly from five trees. We took the figs back to the laboratory, and the female flowers (about 10 g in weight) were carefully excised with a razor blade. The female flowers were immediately frozen in liquid nitrogen and stored at − 80 °C for further use.

### Sample preparation and metabolite extraction

Fig female flower samples were further grounded and thoroughly mixed with a mortar and pestle in liquid nitrogen. The freeze-dried tissue was crushed using a mixer mill (MM 400, Retsch) with Zirconia beads for 1.5 min at 30 Hz. The powdered sample (100 mg) was weighed and extracted overnight at 4 °C with 1.0 mL 70% aqueous methanol. Following centrifugation at 10,000×*g* for 10 min, the extracts were absorbed (CNWBOND Carbon-GCB SPE Cartridge, 250 mg, 3 mL; ANPEL, Shanghai, China, www.anpel.com.cn/cnw) and filtrated (SCAA-104, 0.22 μm pore size; ANPEL) before LC-MS analysis.

The fig female flower sample extracts were analyzed using an LC-ESI-MS/MS system (HPLC, Shim-pack UFLC SHIMADZU CBM30A system, www.shimadzu.com.cn; MS, Applied Biosystems 6500 Q TRAP, www.appliedbiosystems.com.cn). The analytical conditions were as follows: HPLC column, Waters ACQUITY UPLC HSS T3 C18 (1.8 μm, 2.1 mm × 100 mm); the solvent system was water (0.04% acetic acid) and acetonitrile (0.04% acetic acid); gradient program, 95:5 V/V at 0 min, 5:95 V/V at 11.0 min, 5:95 V/V at 12.0 min, 95:5 V/V at 12.1 min, 95:5 V/V at 15.0 min; flow rate, 0.40 ml/min; temperature, 40 °C; injection volume, 2 μL. The effluent was alternatively connected to an ESI-triple quadrupole-linear ion trap (Q TRAP)-MS [42].

### Metabolite identification and quantification

LIT and triple quadrupole (QQQ) scans were acquired on a QQQ-linear ion trap mass spectrometer (Q TRAP), API 6500 Q TRAP LC/MS/MS system, equipped with an ESI Turbo Ion-Spray interface, operating in a positive ion mode and controlled by Analyst 1.6.3 software (AB Sciex). The ESI source operation parameters were as follows: ion source, turbo spray; source temperature, 500 °C; ion spray voltage (IS) 5500 V; ion source gas I (GSI), gas II (GSII), curtain gas (CUR) were set at 55, 60, and 25.0 psi, respectively; the collision gas (CAD) was high. Instrument tuning and mass calibration were performed with 10 and 100 μmol/L polypropylene glycol solutions in QQQ and LIT modes, respectively. QQQ scans were acquired as MRM experiments with collision gas (nitrogen) set to 5 psi. Declustering potentials (DP) and collision energies (CE) for individual MRM transitions were done with further DP and CE optimization. A specific set of MRM transitions were monitored for each period according to the metabolites eluted within this period. Metabolite data analysis was conducted with Analyst 1.6.1 software (AB SCIEX, Ontario, Canada). Metabolite quantification was carried out using MRM. Partial least squares discriminant analysis (PLS-DA) was carried out with the metabolites identified. Metabolites with significant differences in content were set with thresholds of variable importance in projection (VIP) ≥ 1 and fold change ≥2 or ≤ 0.5 [43, 44].

### RNA-seq and annotation

Six libraries representing the two female flower samples and the three replicates were constructed for transcriptome sequencing. Total RNA extraction from fig material was performed using the CTAB method [45]. RNA concentration and purity were measured by NanoDrop 2000 (NanoDrop Technologies, Wilmington, DE, USA) and the Agilent Bioanalyzer 2100 system (Agilent Technologies, Palo Alto, CA, USA), respectively. After RNA integrity was determined by 1% agarose gel electrophoresis, RNA concentration was adjusted to the same level. mRNA was isolated from total RNA using magnetic beads with oligo (dT); cDNA was synthesized using a cDNA Synthesis Kit (TaKaRa, Japan) and linking the sequencing adapter to both ends [46]. The library preparations were sequenced on an Illumina HiSeq 4000 platform and the unigene sequences obtained from our laboratory transcriptome database by RSEM software were integrated for annotation [29].

### Transcriptome data analysis

Raw reads were processed with FastQC (http://www.bioinformatics.babraham.ac.uk/projects/fastqc) to filter out adapters and low-quality sequences. For gene expression analysis, counts were mapped to the reading of each gene by HTSeq (v0.5.4p3) and then normalized to fragments per kilobase of transcript per million mapped reads (FPKM). EdgeR software (http://www.bioconductor.org/packages/2.12/bioc/html/edgeR.html) was used to analyze differentially expressed genes (DEGs). Screening of significant DEGs was performed with *p*-value (p-FDR) ≤ 0.05 and |log2FC| ≥ 1 as the criteria. Enrichment analyses were performed using GOatools (https://github.com/tanghaibao/GOatools) and Fisher’s exact test. To control the calculated false positive rate, the *p*-values were corrected using four multiple test methods, and significant differences in GO functionalization were performed on the differential genes at *p* ≤ 0.05. KEGG pathway enrichment analysis was performed using KOBAS software (http://kobas.cbi.pku.edu.cn/home.do) with a corrected *P*-value ≤0.05 [45].

### Hormone measurement and RT-qPCR verification

The levels of auxin (IAA), gibberellin (GA), zeatin (ZR) and abscisic acid (ABA) in fig female flower tissues were determined by enzyme-linked immunoassay (ELISA) [47]. The enzyme immunoassay kit used for the assay was purchased from Yunnan Agricultural University, and the hormone assay tests were performed according to the described steps, and each sample was repeated three times. Finally, the mean values were calculated, and significance analysis was performed using SPSS 17.0 software.

Based on the transcriptome data of CK and BF fig female flowers, the expression level of 15 DEGs were validated. The PCR was performed with an ABI 7500 Fast Real-Time Detection System (Applied Biosystems) using the Ultra SYBR Mix kit (TaKaRa, Japan). The amplification system consisted of 10 μL Ultra SYBR Premix System II, 0.5 μL of 10 μmol/L upstream primer, 0.5 μL of 10 μmol/L downstream primer, 2 μL template, and double distilled water to a total volume of 20 μL. The amplification program was 95 °C for 10 min, followed by 40 cycles of 95 °C for 5 s and 58 °C for 30 s. Relative quantitative analysis of data was performed by the 2^−ΔΔCT^ method with β-actin as the reference gene. The primers used for RT-qPCR are listed as Additional file 1: Table S1.

## Supplementary Information

**Additional file 1.**

## Data Availability

The datasets generated and analyzed in the current study are available from the corresponding author on reasonable request. All data generated or analyzed during this study are included in this published article and its Supplementary information files. The raw RNA-seq data are freely available at: www.ncbi.nlm.nih.gov/bioproject/ PRJNA494945.

## Abbreviations

4CL: 4-Coumarate:coenzyme A ligase
ANS: Anthocyanidin synthase
bHLH: Basic helix-loop-helix
CHI: Chalcone isomerase
CHS: Chalcone synthase
COG: Clusters of orthologous groups of proteins database
DEG: Differentially expressed gene
DFR: Dihydroflavonol 4-reductase
FPKM: Fragments per kilobase of exon model per million mapped reads
F3H: Flavanone 3-hydroxylase
F3′H: Flavanone 3′-hydroxylase
GO: Gene Ontology
KEGG: Kyoto Encyclopedia of Genes and Genomes
LAR: Leucoanthocyanidin reductase
MYB: v-myb avian myeloblastosis viral oncogene homolog
PAL: Phenylalanine ammonia-lyase
UFGT: UDP-glucose: flavonoid 3-O-glucosyltransferase
