# Supplementary material for: Metabolome and transcriptome analysis of flavor components and flavonoid biosynthesis in fig female flower tissues (Ficus carica L.) after bagging
Source: BMC Plant Biol. 2021 Aug 25;21:396. doi: 10.1186/s12870-021-03169-1 (PMC8386004; doi:10.1186/s12870-021-03169-1)
Supplement: Supplementary file 1 — Additional file 1. [file 12870_2021_3169_MOESM1_ESM.pdf]

Metabolome and Transcriptome analysis of flavor components and flavonoid biosynthesis in fig fruit (*Ficus carica* L.) after bagging

Ziran Wang[ab], Miaoyu Song[b], Zhe Wang[b], Shangwu Chen[c], Huiqin Ma[b*]

[a] College of Horticulture and Landscape, Yunnan Agricultural University, Kunming, 650224, PR China

[b] College of Horticulture, China Agricultural University, Beijing, 100193, PR China

[c] College of Food Science and Nutritional Engineering, China Agricultural University, Beijing, 100193, PR China

Ziran Wang: wangziran@cau.edu.cn

Miaoyu Song: songmiaoyu@cau.edu.cn

Zhe Wang: wangzhe2018@cau.edu.cn

Shangwu Chen: swchen@cau.edu.cn

Huiqin Ma: hqma@cau.edu.cn

*Correspondence: Huiqin Ma, hqma@cau.edu.cn

Table S1. Primer sequences of genes used for verification of digital gene-expression results by RT-qPCR.

Gene Name	**Seq ID**	**Forward Primer (5’-3’)**	**Reverse Primer (5’-3’)**
Actin	Reference gene	GCCATTCAAGCCGTGCTTT	TGGGAACAGTGTGGCTGACA
FcMYB-1	c66005_g2	TCCATCTTGTCAGCAGCAAC	GCCGAGGAAATCATAACCAA
FcMYB-2	c43844_g1	CGTAGGGTATCCCGGAATTT	CAACCTTCTCATCCCCAAGA
FcbHLH-1	c66005_g2	TCCATCTTGTCAGCAGCAAC	GCCGAGGAAATCATAACCAA
FcbHLH-2	c39798_g1	CAAAAAGGCTACACCGAAGC	GTTTCGTAGATGGCCGGTTA
FcPAL	c388_g2	CTAGGTGGCTCCCCTTGAGT	TTTGCCTTTTTGCCATTTTC
FcC4H	c39884_g1	AGTCATGGACGCGTAAAACC	CTGGAATCATCATCGCATTG
FcCHS	c46769_g3	CAAAGCACGTGACTCCCATC	ATTTCCTCGACGGTCACCAT
FcCHI	c46816_g1	CACTTCCCTTCTTCCCCTTC	TGGTTGATGAAATCCCCTTC
FcDFR	c18574_g2	AGGTGGAGGTAAGAGCAGCA	GATAGCCCATGCCACAACTT
FcANS-1	c23651_g1	TCACCCTCAGACAAGCTCCT	ACCGACCAATTGCAGAAAAG
FcUFGT-1	c45009_g5	GAGTTGATGGTGGGGCTTTC	TCACTCTTGAACTCCTCCGG
FcGA2ox	c32275_g2	TCGAGCATCGTCATGAACTC	TTCTGTCTCAGCCAGCATTG
FcNCED	c36086_g2	GAGGTAGACGCCACGTAAGC	CCCGGTTCAGAGAGTAGCAG
FcABA 8'-h	c23609_g1	TGGGATGCTTGTTGAGTTCG	ACGGCTGGTTAGTTTCCTCA
FcARF	c32996_g1	GGGAACTACCGCAACACAGT	CAGGCCCCCTTTATTTGATT

Table S2. The classification of compounds detected by the metabolome is in CK and BF.

Index	Compounds	CK	BF	VIP	Fold_Change	Log2FC
**Alcohols**						
pme2639	Enterodiol	2.33E+04	1.57E+04	1.55E+00	6.72E-01	-5.73E-01
pma2987	Histidinol	2.46E+07	2.32E+07	1.44E+00	9.43E-01	-8.45E-02
pmf0388	Dehydrovomifoliol	9.39E+03	8.51E+03	1.23E+00	9.06E-01	-1.42E-01
pmb0767	D-erythro-Dihydrosphingosine	2.93E+05	4.22E+05	1.21E+00	1.44E+00	5.25E-01
pme0516	Inositol	4.03E+05	4.52E+05	1.09E+00	1.12E+00	1.66E-01
pmb0764	4-Methyl-5-thiazoleethanol	5.17E+04	4.17E+04	1.01E+00	8.07E-01	-3.09E-01
pme2134	DL-threitol	1.62E+06	1.68E+06	9.62E-01	1.04E+00	6.06E-02
pmf0175	2-Decanol	1.10E+07	1.01E+07	7.81E-01	9.18E-01	-1.23E-01
pmf0256	3-Methyl-1-pentanol	6.15E+04	5.57E+04	7.61E-01	9.06E-01	-1.43E-01
pmf0348	2,6-Dimethyl-7-octene-2,3,6-triol	1.16E+05	1.01E+05	6.97E-01	8.72E-01	-1.97E-01
pmf0174	1-Decanol	1.00E+07	9.41E+06	6.44E-01	9.38E-01	-9.16E-02
pme2529	1,5-Anhydro-D-glucitol	1.12E+05	1.18E+05	6.38E-01	1.06E+00	7.84E-02
pme3081	4-(Aminomethyl)-5-(hydroxymethyl)-2-methylpyridin-3-ol	1.56E+04	2.01E+04	6.36E-01	1.29E+00	3.68E-01
pme2256	D-Arabitol	8.42E+03	7.77E+03	2.77E-01	9.23E-01	-1.15E-01
pme1894	D-Sorbitol	9.41E+06	9.25E+06	3.24E-02	9.83E-01	-2.43E-02

**Alkaloids**						
pmb0782	Piperidine	4.17E+05	5.36E+05	1.57E+00	1.28E+00	3.62E-01
pma1840	Coumaroyl choline	3.48E+04	5.39E+04	1.50E+00	1.55E+00	6.30E-01
pmf0267	Indole-3-carboxylic	3.70E+05	2.76E+05	1.46E+00	7.45E-01	-4.24E-01
pmf0269	Tryptophol	4.68E+04	3.35E+04	1.38E+00	7.16E-01	-4.82E-01
pmf0557	Acid orange 20	4.69E+04	2.98E+04	1.37E+00	6.35E-01	-6.55E-01
pmf0559	Abrine	1.74E+05	1.63E+05	1.31E+00	9.41E-01	-8.83E-02
pme3333	1,4-dihydro-1-Methyl-4-oxo-3-pyridinecarboxamide	1.81E+05	2.32E+05	1.31E+00	1.28E+00	3.59E-01
pmb4486	Hordatine B	8.71E+03	1.30E+04	1.30E+00	1.49E+00	5.78E-01
pmf0327	lumichrome	1.07E+05	1.38E+05	1.29E+00	1.28E+00	3.60E-01
pme1691	Acetylcholine	6.03E+05	8.04E+05	1.28E+00	1.33E+00	4.15E-01
pmf0336	Indole-3-carboxylic acid	2.69E+05	2.04E+05	1.27E+00	7.59E-01	-3.97E-01
pme2786	N-Acetyl-5-hydroxytryptamine	5.41E+05	6.48E+05	1.26E+00	1.20E+00	2.60E-01
pme2594	4-Pyridoxic acid	6.10E+04	5.16E+04	1.12E+00	8.46E-01	-2.42E-01
pme1450	Melatonin	8.09E+03	1.10E+04	1.08E+00	1.36E+00	4.41E-01
pme2268	Trigonelline	1.55E+07	1.72E+07	9.97E-01	1.11E+00	1.51E-01
pmf0311	Coronatine	7.06E+04	3.47E+04	9.81E-01	4.91E-01	-1.03E+00
pmf0455	Peimine	3.17E+05	1.89E+05	9.56E-01	5.95E-01	-7.49E-01
pmb0037	Hordenine	2.19E+07	1.75E+07	9.50E-01	7.99E-01	-3.24E-01

pme1738	3-Carbamyl-1-methylpyridinium (1-Methylnicotinamide)	1.19E+04	9.10E+03	9.32E-01	7.68E-01	-3.81E-01
pme2024	serotonin	3.65E+06	3.12E+06	9.21E-01	8.54E-01	-2.28E-01
pmb0774	N-hydroxy tryptamine	2.70E+06	2.36E+06	9.16E-01	8.74E-01	-1.94E-01
pme1417	L-Tryptamine	5.67E+06	5.24E+06	8.61E-01	9.23E-01	-1.15E-01
pmf0323	(-)-Cotinine	6.12E+04	5.36E+04	7.76E-01	8.77E-01	-1.89E-01
pma2405	Feruloylcholine	2.81E+04	2.48E+04	7.15E-01	8.83E-01	-1.80E-01
pmf0052	Sophoridine	2.30E+04	2.13E+04	6.69E-01	9.26E-01	-1.12E-01
pmb0484	Choline	7.67E+06	6.95E+06	5.48E-01	9.05E-01	-1.44E-01
pma6270	sn-Glycero-3-phosphocholine	6.62E+05	7.56E+05	5.29E-01	1.14E+00	1.91E-01
pmf0403	L-Hyoscyamine	9.94E+04	9.17E+04	5.23E-01	9.23E-01	-1.16E-01
pme1828	Betaine	1.36E+06	1.40E+06	5.05E-01	1.03E+00	4.65E-02
pme2108	L-Carnitine	1.23E+05	1.15E+05	4.99E-01	9.39E-01	-9.12E-02
pmf0384	Nicotine	5.17E+04	6.28E+04	4.87E-01	1.21E+00	2.78E-01
pmf0565	L-Dencichin	7.54E+04	7.00E+04	4.86E-01	9.29E-01	-1.06E-01
pmb0785	Isoquinoline	2.38E+04	2.49E+04	3.13E-01	1.05E+00	6.84E-02
pmb1754	O-Phosphocholine	8.97E+05	9.21E+05	2.50E-01	1.03E+00	3.73E-02
pmf0531	N-Methylcytisine	4.31E+04	4.92E+04	1.85E-01	1.14E+00	1.89E-01
pmf0378	Asimilobine	6.71E+03	6.10E+03	1.83E-01	9.10E-01	-1.37E-01
pme2155	Theobromine	4.51E+03	4.42E+03	1.73E-01	9.80E-01	-2.90E-02

pmb2211	Cocamidopropyl betaine	3.73E+04	3.98E+04	1.37E-02	1.07E+00	9.13E-02
pmb0426	5-Methoxy-N,N-dimethyltryptamine	1.44E+05	1.42E+05	5.91E-03	9.89E-01	-1.65E-02
**Amino acid and derivatives**						
pme1002	L-Tyramine	2.01E+05	7.84E+05	1.40E+00	3.90E+00	1.96E+00
pme0164	N-γ-Acetyl-N-2-Formyl-5-methoxykynurenamine	2.63E+05	4.23E+05	1.47E+00	1.61E+00	6.85E-01
pme0042	L-(+)-Arginine	1.03E+05	1.64E+05	8.68E-01	1.59E+00	6.67E-01
pme0026	L-(+)-Lysine	1.19E+06	1.83E+06	8.30E-01	1.53E+00	6.15E-01
pme2142	L-Theanine	1.83E+05	2.76E+05	1.32E+00	1.51E+00	5.95E-01
pmb0962	Lysine butyrate	1.83E+04	2.77E+04	9.49E-01	1.51E+00	5.95E-01
pme0120	5-Aminovaleric acid	5.41E+04	8.15E+04	6.69E-01	1.51E+00	5.92E-01
pme0025	L-Kynurenine	6.31E+04	9.29E+04	1.29E+00	1.47E+00	5.57E-01
pmb2857	L-Glutamic acid O-glucoside	2.39E+04	3.34E+04	1.24E+00	1.40E+00	4.82E-01
pme0113	γ-Glu-Cys	1.17E+05	1.61E+05	1.25E+00	1.38E+00	4.60E-01
pme3388	H-HomoArg-OH	9.86E+04	1.34E+05	1.00E+00	1.36E+00	4.42E-01
pme0231	N-(3-Indolylacetyl)-L-alanine	1.06E+05	1.40E+05	1.25E+00	1.32E+00	3.97E-01
pmb2591	Acetyl tryptophan	3.48E+05	4.57E+05	1.06E+00	1.31E+00	3.92E-01
pme0170	Nα-Acetyl-L-arginine	1.91E+05	2.47E+05	1.01E+00	1.30E+00	3.75E-01
pme0009	L-Serine	1.26E+06	1.62E+06	1.13E+00	1.29E+00	3.66E-01
pme1712	L-Saccharopine	1.17E+05	1.50E+05	8.42E-01	1.28E+00	3.51E-01

pme0174	N-Propionylglycine	9.65E+05	1.19E+06	1.53E+00	1.23E+00	3.02E-01
pme0190	N-Glycyl-L-leucine	2.99E+04	3.64E+04	8.66E-01	1.21E+00	2.80E-01
pme1286	S-(5'-Adenosy)-L-homocysteine	1.75E+05	2.12E+05	8.82E-01	1.21E+00	2.72E-01
pmf0484	alpha-Aminocaproic acid	5.37E+06	6.48E+06	1.33E+00	1.21E+00	2.69E-01
pme1228	5-Hydroxy-L-tryptophan	1.55E+05	1.84E+05	1.25E+00	1.19E+00	2.52E-01
pmb0449	2-Aminoadipic acid (L-Homoglutamic acid)	1.68E+06	1.99E+06	1.49E+00	1.19E+00	2.46E-01
pme0124	Glycyl-L-proline	1.11E+05	1.31E+05	1.11E+00	1.18E+00	2.37E-01
pme3030	N-Acetylcysteine	8.62E+04	1.01E+05	8.17E-01	1.18E+00	2.33E-01
pmf0448	S-Allyl-L-cysteine	3.44E+04	4.03E+04	5.64E-01	1.17E+00	2.28E-01
pme1419	L-Methionine methyl ester	5.58E+05	6.53E+05	1.15E+00	1.17E+00	2.27E-01
pme3033	N,N-Dimethylglycine	1.72E+06	1.99E+06	8.24E-01	1.15E+00	2.08E-01
pme0075	N-Acetyl-L-glutamic acid	8.73E+04	1.01E+05	1.07E+00	1.15E+00	2.07E-01
pme2743	N-Phenylacetylglycine	3.93E+04	4.52E+04	1.17E+00	1.15E+00	2.04E-01
pme0050	L-Tryptophan	1.52E+07	1.74E+07	1.39E+00	1.14E+00	1.94E-01
pme2914	3-Hydroxy-3-methylpentane-1,5-dioic acid	1.37E+07	1.56E+07	1.15E+00	1.14E+00	1.90E-01
pme0020	L-Phenylalanine	1.53E+07	1.74E+07	8.24E-01	1.14E+00	1.84E-01
pme0161	L-Homoserine	1.46E+05	1.64E+05	1.31E+00	1.13E+00	1.71E-01
pmf0594	D-(+)-Phenylalanine	1.32E+06	1.48E+06	7.55E-01	1.12E+00	1.68E-01
pme1313	N'-Formylkynurenine	1.10E+05	1.23E+05	7.75E-01	1.12E+00	1.64E-01

pmf0018	1-Aminocyclopropanecarboxylic acid	1.12E+05	1.25E+05	4.28E-01	1.12E+00	1.61E-01
pme1104	L-Isoleucine	8.29E+04	9.23E+04	1.27E+00	1.11E+00	1.55E-01
pme0195	L-Cysteine	4.15E+04	4.62E+04	4.32E-01	1.11E+00	1.55E-01
pmf0292	D-erythro-sphinganine	4.38E+04	4.87E+04	3.48E-01	1.11E+00	1.53E-01
pme0030	L-(-)-Tyrosine	2.07E+06	2.27E+06	9.26E-01	1.10E+00	1.36E-01
pme1408	L-Glutamine	1.62E+06	1.76E+06	7.14E-01	1.08E+00	1.12E-01
pme2758	4-Hydroxy-L-glutamic acid	2.76E+04	2.96E+04	4.48E-01	1.07E+00	1.03E-01
pme0036	L-Histidine	5.95E+05	6.37E+05	1.25E+00	1.07E+00	9.85E-02
pme2427	N-Acetyl-L-tyrosine	3.88E+04	4.14E+04	4.41E-01	1.07E+00	9.64E-02
pme1090	Glutathione reduced form	1.21E+07	1.30E+07	6.00E-01	1.07E+00	9.51E-02
pme0018	L-Leucine	2.05E+05	2.16E+05	5.23E-01	1.06E+00	7.76E-02
pme2122	Histamine	3.02E+04	3.16E+04	5.08E-01	1.05E+00	6.49E-02
pme0039	L-Valine	1.00E+07	1.04E+07	1.05E+00	1.04E+00	5.21E-02
pme2617	Methionine sulfoxide	1.02E+07	1.06E+07	5.43E-01	1.03E+00	4.91E-02
pme0004	L-Homocitrulline	1.43E+05	1.48E+05	4.98E-02	1.03E+00	4.72E-02
pmb0464	Aspartic acid di-O-glucoside	2.61E+07	2.68E+07	3.83E-01	1.03E+00	4.16E-02
pme0181	1-Methylhistidine	1.42E+05	1.46E+05	4.65E-01	1.03E+00	3.72E-02
pme0006	L-Proline	1.84E+07	1.89E+07	4.86E-01	1.02E+00	3.56E-02
pme2634	Dl-Norvaline	9.40E+06	9.63E+06	6.03E-01	1.02E+00	3.50E-02

pme0226	L-Asparagine	1.04E+07	1.06E+07	1.00E-01	1.02E+00	3.23E-02
pme1210	L-Methionine	1.14E+07	1.16E+07	2.13E-01	1.02E+00	2.45E-02
pme2046	3-N-Methyl-L-histidine	3.59E+04	3.63E+04	6.77E-02	1.01E+00	1.71E-02
pme2698	Phe-Phe	4.52E+04	4.54E+04	4.22E-03	1.00E+00	4.28E-03
pmb0115	3-Hydroxykynurenine	4.93E+04	4.91E+04	7.80E-02	9.97E-01	-5.05E-03
pme3384	N-Acetylthreonine	2.20E+05	2.20E+05	1.14E-02	9.96E-01	-5.11E-03
pme0023	L-Threonine	3.23E+06	3.21E+06	1.12E-01	9.96E-01	-6.35E-03
pmf0441	N-Acetyl-L-phenylalanine	1.83E+05	1.82E+05	1.48E-02	9.95E-01	-7.39E-03
pmf0016	DL-Methionine	7.36E+06	7.28E+06	8.08E-02	9.89E-01	-1.57E-02
pme0252	N-Acetyl-l-leucine	2.88E+05	2.78E+05	4.58E-01	9.66E-01	-4.94E-02
pmf0593	D-(-)-Valine	1.60E+06	1.54E+06	4.85E-01	9.64E-01	-5.32E-02
pme3351	Allysine(6-Oxo DL-Norleucine)	2.37E+04	2.26E+04	3.41E-01	9.51E-01	-7.20E-02
pme1988	L-Alanine	1.55E+05	1.47E+05	4.45E-01	9.47E-01	-7.82E-02
pmb3264	Glutathione oxidized	4.95E+05	4.67E+05	4.59E-01	9.45E-01	-8.18E-02
pme3038	5-oxoproline	5.90E+07	5.54E+07	1.18E+00	9.40E-01	-8.96E-02
pme2735	S-(5'-Adenosyl)-L-methionine	9.10E+05	8.54E+05	2.60E-01	9.39E-01	-9.04E-02
pmb2561	N-Acetylmethionine	1.34E+05	1.26E+05	1.25E+00	9.39E-01	-9.15E-02
pmb2873	3-(2-Naphthyl)-D-alanine	1.51E+05	1.41E+05	3.50E-01	9.37E-01	-9.35E-02
pme0278	2,6-Diaminooimelic acid	1.82E+05	1.67E+05	5.84E-01	9.18E-01	-1.24E-01

pme1239	S-(methyl)glutathione	2.52E+04	2.30E+04	3.94E-01	9.12E-01	-1.33E-01
pme0013	L-Glutamic acid	5.31E+06	4.82E+06	8.21E-01	9.08E-01	-1.39E-01
pme2569	(5-L-Glutamyl)-L-amino acid	6.53E+04	5.90E+04	6.15E-01	9.03E-01	-1.47E-01
pmf0589	Glutamic acid	5.21E+06	4.70E+06	7.20E-01	9.02E-01	-1.49E-01
pme0116	L-Carnosine	1.61E+04	1.44E+04	7.09E-01	8.97E-01	-1.57E-01
pmf0585	Proline	1.29E+07	1.15E+07	9.20E-01	8.93E-01	-1.64E-01
pmb2855	L-Glutamine O-hexside	1.13E+05	1.00E+05	4.71E-01	8.92E-01	-1.65E-01
pmb0468	N-formylmethionine	1.30E+07	1.12E+07	1.02E+00	8.60E-01	-2.18E-01
pme3194	N-acetylglycine	1.06E+06	8.81E+05	1.02E+00	8.34E-01	-2.63E-01
pme3017	2-Aminoisobutyric acid	2.14E+05	1.76E+05	9.15E-01	8.24E-01	-2.79E-01
pme2527	L(+)-Ornithine	1.63E+05	1.34E+05	1.20E+00	8.24E-01	-2.79E-01
pme0137	Nα-Acetyl-L-glutamine	2.04E+05	1.68E+05	1.45E+00	8.22E-01	-2.83E-01
pme0118	Pyrrole-2-carboxylic acid	3.75E+04	3.08E+04	1.38E+00	8.22E-01	-2.83E-01
pme0066	Guanidineacetic acid	1.48E+05	1.20E+05	7.55E-01	8.14E-01	-2.97E-01
pme1368	L-Pipecolic acid	5.70E+04	4.52E+04	9.20E-01	7.94E-01	-3.33E-01
pme0008	L-Citrulline	1.58E+06	1.25E+06	1.09E+00	7.91E-01	-3.38E-01
pme0132	Asp-phe	7.33E+04	5.79E+04	1.26E+00	7.91E-01	-3.39E-01
pme0056	2,3-dimethylsuccinic acid	6.05E+05	4.78E+05	9.60E-01	7.90E-01	-3.41E-01
pme0015	L-(-)-Cystine	2.76E+04	2.12E+04	7.27E-01	7.71E-01	-3.76E-01

pme0122	N6-Acetyl-L-lysine	1.79E+05	1.37E+05	1.47E+00	7.66E-01	-3.85E-01
pme2559	N-Acetylaspartate	3.01E+05	2.14E+05	1.39E+00	7.10E-01	-4.93E-01
pme0011	L-Aspartic acid	1.19E+07	7.10E+06	1.07E+00	5.98E-01	-7.42E-01
pme3179	CYS-GLY	5.61E+04	3.25E+04	1.49E+00	5.79E-01	-7.88E-01
pme2890	L-Homocystine	2.09E+05	1.19E+05	1.28E+00	5.72E-01	-8.05E-01
pme0177	Phenylacetyl-L-glutamine	1.61E+05	9.01E+04	1.06E+00	5.61E-01	-8.33E-01
pmf0587	Aspartic acid	1.05E+07	5.83E+06	1.15E+00	5.55E-01	-8.49E-01
**Anthocyanins**						
pmf0203	Peonidin 3-O-glucoside chloride	6.39E+05	8.43E+05	1.56E+00	1.32E+00	4.00E-01
pma1590	Peonidin O-hexoside	5.88E+05	7.85E+05	1.49E+00	1.34E+00	4.17E-01
pmb0542	Cyanidin 3-O-malonylhexoside	2.91E+05	6.08E+05	1.46E+00	2.09E+00	1.06E+00
pmb2959	Cyanidin O-acetylhexoside	1.91E+05	4.13E+05	1.45E+00	2.16E+00	1.11E+00
pme1773	Cyanidin 3-O-rutinoside (Keracyanin)	5.16E+07	6.68E+07	1.44E+00	1.29E+00	3.72E-01
pme1777	Cyanidin 3,5-O-diglucoside (Cyanin)	4.87E+07	7.81E+07	1.43E+00	1.60E+00	6.82E-01
pmb2957	Cyanidin O-syringic acid	3.62E+06	5.51E+06	1.42E+00	1.52E+00	6.07E-01
pme1397	Pelargonidin	7.52E+04	5.19E+04	1.24E+00	6.91E-01	-5.34E-01
pme0094	Cyanidin 3-O-glucoside (Kuromanin)	5.50E+05	6.29E+05	1.23E+00	1.14E+00	1.93E-01
pme3392	Pelargonidin 3-O-beta-D-glucoside（Callistephin chloride)	3.65E+05	4.41E+05	1.22E+00	1.21E+00	2.70E-01
pme1793	Pelargonin	2.40E+05	2.89E+05	1.17E+00	1.20E+00	2.67E-01

pmb2962	Pelargonidin O-acetylhexoside	1.77E+04	2.01E+04	9.88E-01	1.14E+00	1.84E-01
pmf0616	Malvidin 3-acetyl-5-diglucoside	4.57E+04	3.92E+04	9.74E-01	8.58E-01	-2.22E-01
pmf0027	Cyanidin 3-O-galactoside	4.64E+07	5.15E+07	9.21E-01	1.11E+00	1.51E-01
pme0443	Malvidin 3-O-galactoside	3.55E+04	3.10E+04	8.45E-01	8.72E-01	-1.98E-01
pmb2961	Peonidin O-malonylhexoside	3.65E+04	3.85E+04	3.01E-01	1.06E+00	7.94E-02
**Carbohydrates**						
pma0134	D(-)-Threose	5.60E+03	2.07E+04	1.03E+00	3.70E+00	1.89E+00
pmf0032	Galactinol	1.24E+06	1.65E+06	1.18E+00	1.33E+00	4.08E-01
pmf0574	Sucralose	4.39E+04	5.19E+04	8.67E-01	1.18E+00	2.42E-01
pme2019	DL-Arabinose	2.09E+05	2.32E+05	9.38E-01	1.11E+00	1.55E-01
pmf0485	Panose	9.83E+04	1.06E+05	7.20E-01	1.08E+00	1.07E-01
pme1684	D-(+)-Glucono-1,5-lactone	2.56E+04	2.75E+04	1.80E-01	1.07E+00	1.00E-01
pme1846	D(+)-Glucose	4.89E+06	5.15E+06	4.51E-01	1.05E+00	7.36E-02
pmf0139	D-(+)-Galactose	7.17E+06	7.48E+06	1.81E-01	1.04E+00	6.22E-02
pmf0138	D-(+)-Mannose	1.13E+07	1.13E+07	6.40E-02	9.99E-01	-1.70E-03
pmb0786	Glucosamine	2.16E+06	2.12E+06	4.11E-01	9.83E-01	-2.53E-02
pmf0282	Melibiose	3.79E+05	3.70E+05	7.53E-02	9.77E-01	-3.30E-02
pme2755	N-Acetyl-D-glucosamine	4.17E+05	4.02E+05	1.64E-01	9.63E-01	-5.39E-02
pmb2653	D(+)-Melezitose O-rhamnoside	4.75E+04	4.47E+04	5.32E-02	9.43E-01	-8.51E-02

pme0524	D(+)-Melezitose	1.50E+05	1.40E+05	5.71E-01	9.29E-01	-1.07E-01
pmb3088	Trehalose 6-phosphate	1.17E+05	1.09E+05	7.09E-01	9.28E-01	-1.07E-01
pmf0220	D-Fructose 6-phosphate-disodium salt	5.53E+06	4.61E+06	1.17E+00	8.34E-01	-2.62E-01
pmf0035	Glucose-1-phosphate	5.63E+06	4.61E+06	1.27E+00	8.19E-01	-2.89E-01
pma6455	Ribulose-5-phosphate	2.35E+05	1.86E+05	1.45E+00	7.92E-01	-3.36E-01
pme3313	D-Fructose 6-phosphate	4.21E+06	3.22E+06	1.40E+00	7.64E-01	-3.89E-01
pme3160	D-Glucose 6-phosphate	7.07E+06	4.93E+06	1.60E+00	6.98E-01	-5.19E-01
**Flavanone**						
pme1598	Hesperetin 5-O-glucoside	1.55E+06	2.18E+06	1.40E+00	1.40E+00	4.89E-01
pme3285	Afzelechin (3,5,7,4'-Tetrahydroxyflavan)	2.38E+04	3.07E+04	1.37E+00	1.29E+00	3.66E-01
pma0791	Naringenin O-malonylhexoside	9.14E+04	1.13E+05	1.36E+00	1.24E+00	3.10E-01
pme2982	Pinocembrin (Dihydrochrysin)	2.19E+04	1.62E+05	1.32E+00	7.38E+00	2.88E+00
pme1201	Phloretin	1.54E+04	2.54E+04	1.20E+00	1.65E+00	7.21E-01
pme0372	Naringenin 7-O-glucoside (Prunin)	2.29E+05	3.34E+05	1.18E+00	1.46E+00	5.49E-01
pme2960	Naringenin chalcone	1.38E+05	2.20E+05	1.13E+00	1.59E+00	6.72E-01
pme0377	Naringenin	1.43E+05	2.34E+05	1.11E+00	1.63E+00	7.03E-01
pme0001	Hesperetin 7-O-neohesperidoside (Neohesperidin)	2.28E+04	2.51E+04	1.08E+00	1.10E+00	1.35E-01
pmf0058	4',5,7-Trihydroxyflavanone	1.37E+05	2.16E+05	1.04E+00	1.58E+00	6.61E-01
pme2950	Hesperetin 7-rutinoside (Hesperidin)	2.39E+04	1.86E+04	9.27E-01	7.79E-01	-3.60E-01

pmc1990	4'-Hydroxy-5,7-dimethoxyflavanone	2.63E+06	3.18E+06	8.93E-01	1.21E+00	2.74E-01
pme2321	Hesperetin	2.95E+04	3.33E+04	8.35E-01	1.13E+00	1.76E-01
pmb2979	Hesperetin O-malonylhexoside	6.05E+04	6.51E+04	5.18E-01	1.07E+00	1.04E-01
pme3461	Homoeriodictyol	4.94E+04	5.08E+04	3.79E-01	1.03E+00	4.01E-02
pme0330	Naringenin 7-O-neohesperidoside (Naringin)	9.31E+04	9.51E+04	1.88E-01	1.02E+00	3.14E-02
pme1580	Eriodictyol	2.28E+05	2.26E+05	9.21E-03	9.89E-01	-1.55E-02
**Flavone**						
pme0379	Apigenin	9.00E+00	1.99E+04	1.69E+00	2.21E+03	1.11E+01
pme0088	Luteolin	4.97E+03	1.02E+04	1.54E+00	2.04E+00	1.03E+00
pmb0605	Apigenin 7-O-glucoside (Cosmosiin)	1.17E+05	3.43E+05	1.53E+00	2.94E+00	1.56E+00
pma0294	Chrysoeriol 5-O-hexoside	1.71E+05	3.60E+05	1.53E+00	2.11E+00	1.08E+00
pme1550	Tangeretin	5.89E+03	2.07E+04	1.53E+00	3.51E+00	1.81E+00
pma6199	Chrysin O-hexoside	1.66E+04	4.24E+04	1.45E+00	2.56E+00	1.36E+00
pmb3023	Eriodictyol C-hexoside	1.87E+05	3.13E+05	1.45E+00	1.67E+00	7.41E-01
pme2459	Luteolin 7-O-glucoside (Cynaroside)	3.35E+05	9.00E+05	1.42E+00	2.69E+00	1.43E+00
pme0374	Isovitexin	2.89E+05	4.32E+05	1.41E+00	1.50E+00	5.84E-01
pme1518	Nobiletin	8.70E+03	3.11E+04	1.39E+00	3.58E+00	1.84E+00
pmb3041	Tricin O-saccharic acid	8.87E+06	8.36E+06	1.37E+00	9.43E-01	-8.52E-02
pme1541	Acacetin	6.46E+04	1.38E+05	1.37E+00	2.13E+00	1.09E+00

pma1087	C-pentosyl-C-hexosyl-apigenin	2.54E+05	3.05E+05	1.37E+00	1.20E+00	2.65E-01
pma6558	Velutin	1.45E+04	3.78E+04	1.36E+00	2.61E+00	1.38E+00
pmb0693	C-hexosyl-apigenin C-pentoside	1.58E+05	1.94E+05	1.36E+00	1.22E+00	2.93E-01
pmb0580	Chrysin 5-O-glucoside (Toringin)	1.68E+04	4.06E+04	1.34E+00	2.42E+00	1.27E+00
pmb0622	C-hexosyl-luteolin O-hexoside	1.50E+05	1.07E+05	1.31E+00	7.09E-01	-4.95E-01
pmb2850	Tricin	6.93E+03	4.83E+03	1.31E+00	6.96E-01	-5.22E-01
pma1108	Apigenin C-glucoside	2.21E+05	3.57E+05	1.30E+00	1.62E+00	6.92E-01
pma6496	Luteolin 6-C-glucoside	1.32E+05	1.97E+05	1.28E+00	1.49E+00	5.75E-01
pme0324	Chrysin	5.59E+03	1.10E+04	1.28E+00	1.97E+00	9.79E-01
pma0825	Chrysin O-malonylhexoside	2.40E+04	3.54E+04	1.26E+00	1.48E+00	5.62E-01
pmb2984	Acetyl-eriodictyol O-hexoside	2.21E+04	3.29E+04	1.26E+00	1.49E+00	5.73E-01
pmb3042	Tricin 5-O-hexoside	4.32E+04	3.63E+04	1.25E+00	8.39E-01	-2.53E-01
pmb3012	Chrysoeriol 7-O-hexoside	9.70E+04	1.73E+05	1.24E+00	1.79E+00	8.36E-01
pme0359	Apigenin 5-O-glucoside	1.63E+05	2.63E+05	1.21E+00	1.61E+00	6.92E-01
pmb2969	Hesperetin C-hexoside O-hexoside	2.85E+04	4.29E+04	1.20E+00	1.50E+00	5.89E-01
pmb2987	Acacetin O-acetyl hexoside	1.30E+05	1.44E+05	1.19E+00	1.11E+00	1.52E-01
pmb0665	Luteolin 8-C-hexosyl-O-hexoside	5.90E+04	8.58E+04	1.18E+00	1.45E+00	5.40E-01
pmb3043	Tricin 5-O-rutinoside	8.81E+03	3.44E+03	1.17E+00	3.90E-01	-1.36E+00
pmb0628	Eriodictiol C-hexosyl-O-hexoside	1.62E+05	2.25E+05	1.16E+00	1.39E+00	4.71E-01

pmf0011	Apigenin 6,8-C-diglucoside	8.38E+04	1.05E+05	1.13E+00	1.25E+00	3.27E-01
pmb0618	8-C-hexosyl-hesperetin O-hexoside	1.89E+06	1.62E+06	1.10E+00	8.56E-01	-2.24E-01
pma6638	O-methylChrysoeriol 7-O-hexoside	1.19E+04	1.92E+04	1.10E+00	1.61E+00	6.91E-01
pmb3024	Luteolin C-hexoside	1.70E+05	2.28E+05	1.09E+00	1.34E+00	4.18E-01
pme3475	Butin	1.46E+05	2.36E+05	1.07E+00	1.62E+00	6.92E-01
pmb0576	Apigenin O-malonylhexoside	5.55E+04	9.34E+04	1.04E+00	1.68E+00	7.50E-01
pma0249	Selgin 5-O-hexoside	3.41E+04	2.93E+04	9.90E-01	8.58E-01	-2.20E-01
pmf0057	4,2',4',6'-Tetrahydroxychalcone	1.20E+05	1.88E+05	9.61E-01	1.56E+00	6.39E-01
pmb0621	C-hexosyl-isorhamnetin O-hexoside	2.23E+04	3.12E+04	9.19E-01	1.40E+00	4.83E-01
pma0760	Selgin O-malonylhexoside	5.92E+04	6.82E+04	8.94E-01	1.15E+00	2.04E-01
pmb3047	Tricin 4'-O-(syringyl alcohol) ether 5-O-hexoside	1.83E+05	1.64E+05	8.93E-01	8.95E-01	-1.60E-01
pme0363	Chrysoeriol	1.26E+04	1.56E+04	8.66E-01	1.24E+00	3.06E-01
pmb0615	Hesperetin C-hexosyl-O-hexosyl-O-hexoside	7.75E+03	3.69E+03	8.53E-01	4.77E-01	-1.07E+00
pme3300	Tricetin	4.90E+03	1.49E+04	8.31E-01	3.03E+00	1.60E+00
pma6371	di-C,C-hexosyl-luteolin	4.81E+04	5.99E+04	7.78E-01	1.24E+00	3.14E-01
pmb0661	Chrysoeriol C-hexosyl-O-rhamnoside	1.83E+05	2.01E+05	7.42E-01	1.10E+00	1.34E-01
pmb0608	Chrysoeriol O-malonylhexoside	1.42E+04	1.12E+04	6.91E-01	7.90E-01	-3.41E-01
pmb0588	Luteolin 3',7-di-O-glucoside	7.58E+04	8.39E+04	6.77E-01	1.11E+00	1.48E-01
pmb0703	6-C-hexosyl-chrysoeriol O-feruloylhexoside	8.67E+03	1.01E+04	6.74E-01	1.17E+00	2.27E-01

pma0724	Naringenin C-hexoside	1.46E+04	1.32E+04	6.70E-01	9.00E-01	-1.52E-01
pmb2954	Luteolin O-hexosyl-O-hexosyl-O-hexoside	2.05E+04	2.17E+04	6.55E-01	1.06E+00	7.81E-02
pme0368	Apigenin 7-rutinoside (Isorhoifolin)	9.49E+04	1.08E+05	6.49E-01	1.14E+00	1.90E-01
pme0333	Apigenin 7-O-neohesperidoside (Rhoifolin)	9.48E+04	8.94E+04	6.22E-01	9.43E-01	-8.41E-02
pmf0005	Narirutin	1.83E+04	2.08E+04	5.95E-01	1.14E+00	1.89E-01
pmb0600	Chrysoeriol 7-O-rutinoside	1.22E+05	1.27E+05	5.70E-01	1.05E+00	6.82E-02
pme3224	Vitexin 2''-O-beta-L-rhamnoside	2.64E+05	2.79E+05	5.56E-01	1.05E+00	7.60E-02
pmb2978	Apigenin O-hexosyl-O-pentoside	1.08E+04	1.01E+04	5.30E-01	9.40E-01	-8.93E-02
pmb2977	Chrysoeriol 8-C-pentosyl-O-rutinoside	1.64E+04	1.48E+04	4.83E-01	9.08E-01	-1.39E-01
pmb1466	Tricin 4'-O-syringic acid	1.11E+04	9.89E+03	4.46E-01	8.92E-01	-1.65E-01
pmb0601	Chrysoeriol O-hexosyl-O-pentoside	2.20E+04	2.38E+04	3.89E-01	1.08E+00	1.13E-01
pmb2996	Velutin O-glucuronic acid	1.56E+04	1.68E+04	3.35E-01	1.08E+00	1.06E-01
pmb0569	Syringetin 5-O-hexoside	1.81E+05	1.72E+05	3.28E-01	9.48E-01	-7.69E-02
pmb0652	C-hexosyl-apigenin O-pentoside	1.81E+04	1.67E+04	2.94E-01	9.23E-01	-1.16E-01
pmb0620	Chrysoeriol 6-C-hexoside 8-C-hexoside-O-hexoside	1.20E+04	1.23E+04	1.57E-01	1.03E+00	3.80E-02
pma0795	Tricetin O-malonylhexoside	3.57E+04	3.49E+04	1.42E-01	9.77E-01	-3.34E-02
pmb0624	6-C-hexosyl-luteolin O-hexoside	9.38E+04	9.33E+04	1.17E-01	9.95E-01	-7.03E-03
pmb0378	Luteolin O-feruloylhexoside	1.92E+03	3.06E+03	9.92E-02	1.59E+00	6.73E-01
pmb0663	8-C-hexosyl-luteolin O-hexoside	1.24E+04	1.30E+04	9.83E-02	1.05E+00	6.93E-02

pmb0631	C-hexosyl-luteolin C-pentoside	7.36E+03	6.10E+03	7.57E-02	8.29E-01	-2.71E-01
pmb0645	6-C-hexosyl-hesperetin O-hexoside	6.07E+04	5.92E+04	2.15E-02	9.75E-01	-3.67E-02
pmb0647	8-C-hexosyl-luteolin O-pentoside	1.89E+04	1.87E+04	1.22E-02	9.88E-01	-1.69E-02
pmf0204	Hyperoside	2.69E+06	3.99E+06	1.38E+00	1.48E+00	5.69E-01
**Flavonoid**						
pmf0208	Isoquercitroside	3.02E+06	4.17E+06	1.36E+00	1.38E+00	4.67E-01
pmf0375	Isorhamnetin 3-O-glucoside	5.39E+04	7.31E+04	1.20E+00	1.36E+00	4.39E-01
pmf0360	Astilbin	1.10E+05	1.47E+05	1.18E+00	1.34E+00	4.21E-01
pmf0247	Orientin	1.18E+05	1.72E+05	1.16E+00	1.46E+00	5.46E-01
pmf0381	Vicenin-3	4.85E+05	5.44E+05	1.15E+00	1.12E+00	1.64E-01
pmf0371	Pedalitin	5.73E+03	3.98E+03	1.00E+00	6.94E-01	-5.28E-01
pmf0278	Gossypitrin	1.99E+04	2.51E+04	8.62E-01	1.26E+00	3.33E-01
pmf0235	Isoschaftoside	3.83E+05	4.15E+05	7.30E-01	1.08E+00	1.15E-01
pmf0471	Apiin	2.04E+04	1.95E+04	7.18E-01	9.53E-01	-7.01E-02
pme1665	Isovitexin 7-O-glucoside (Saponarin)	7.27E+04	6.90E+04	6.78E-01	9.50E-01	-7.40E-02
pmf0369	Persicoside	6.12E+04	5.84E+04	5.95E-01	9.55E-01	-6.63E-02
pmf0362	Hydroxygenkwanin	6.33E+03	8.80E+03	5.87E-01	1.39E+00	4.76E-01
pmf0179	Narcissoside	3.53E+04	3.81E+04	5.65E-01	1.08E+00	1.10E-01
pmf0275	Herbacetin	6.61E+04	6.71E+04	3.89E-01	1.01E+00	2.12E-02

pmf0548	Schaftoside	7.55E+05	7.75E+05	3.38E-01	1.03E+00	3.72E-02
pmf0109	3-O-Acetylpinobanksin	1.71E+04	2.40E+04	3.37E-01	1.40E+00	4.87E-01
pmf0550	Diosmin	1.35E+05	1.32E+05	1.88E-01	9.75E-01	-3.66E-02
pmf0583	Phloridzin	2.22E+05	2.22E+05	1.09E-01	1.00E+00	1.52E-03
pmf0265	5,7-Dihydroxychromone	7.24E+03	7.35E+03	5.73E-02	1.01E+00	2.06E-02
**Flavonol**						
pmb3894	Di-O-methylquercetin	9.30E+05	6.18E+05	1.53E+00	6.65E-01	-5.89E-01
pme3296	Kaempferol 3-O-rhamnoside (Kaempferin)	1.20E+04	4.00E+04	1.48E+00	3.34E+00	1.74E+00
pme3267	Kaempferol 3-O-galactoside (Trifolin)	2.10E+05	4.75E+05	1.45E+00	2.27E+00	1.18E+00
pme1500	Kumatakenin	2.33E+04	5.93E+04	1.41E+00	2.55E+00	1.35E+00
pme0197	Quercetin 3-O-rutinoside (Rutin)	8.79E+05	1.30E+06	1.41E+00	1.48E+00	5.67E-01
pme1506	Kaempferol 7-O-rhamnoside	1.78E+04	5.28E+04	1.41E+00	2.96E+00	1.57E+00
pmb3026	Quercetin O-acetylhexoside	1.06E+05	1.81E+05	1.39E+00	1.71E+00	7.75E-01
pmb0711	Quercetin 7-O-rutinoside	1.55E+06	2.32E+06	1.38E+00	1.50E+00	5.81E-01
pme1521	Dihydroquercetin (Taxifolin)	1.62E+05	1.98E+05	1.38E+00	1.22E+00	2.87E-01
pmb0706	Quercetin 5-O-malonylhexosyl-hexoside	2.46E+05	2.79E+05	1.37E+00	1.13E+00	1.80E-01
pma0214	methylQuercetin O-hexoside	8.04E+04	1.05E+05	1.32E+00	1.31E+00	3.87E-01
pme3211	Quercetin 3-O-glucoside (Isotrifoliin)	2.25E+06	3.03E+06	1.32E+00	1.35E+00	4.28E-01
pme3130	Quercetin 4'-O-glucoside (Spiraeoside)	6.64E+04	1.05E+05	1.31E+00	1.58E+00	6.59E-01

pme2895	Dihydromyricetin	2.95E+04	2.38E+04	1.19E+00	8.06E-01	-3.10E-01
pme3514	Morin	3.29E+05	7.77E+05	1.14E+00	2.36E+00	1.24E+00
pma2088	Quercetin-3,4'-O-di-beta-glucopyranoside	1.32E+04	8.96E+03	1.10E+00	6.80E-01	-5.55E-01
pmb0604	Kaempferol 3-O-glucoside (Astragalin)	4.14E+05	5.06E+05	1.07E+00	1.22E+00	2.90E-01
pme2963	Aromadedrin (Dihydrokaempferol)	6.19E+04	8.16E+04	1.01E+00	1.32E+00	3.99E-01
pme2954	Quercetin	3.09E+04	6.59E+04	9.75E-01	2.13E+00	1.09E+00
pme3407	Laricitrin	1.18E+04	1.40E+04	8.85E-01	1.19E+00	2.49E-01
pmb0709	Quercetin 7-O-malonylhexosyl-hexoside	2.79E+04	3.33E+04	8.18E-01	1.19E+00	2.51E-01
pma6639	Isorhamnetin O-hexoside	1.01E+05	1.17E+05	7.94E-01	1.16E+00	2.13E-01
pme3484	Myricetin 3-O-galactoside	3.08E+04	2.63E+04	7.43E-01	8.54E-01	-2.27E-01
pme1478	Myricetin	1.43E+04	1.25E+04	7.37E-01	8.71E-01	-1.99E-01
pmb0595	Isorhamnetin 5-O-hexoside	1.04E+05	9.96E+04	7.08E-01	9.55E-01	-6.70E-02
pme2493	Kaempferol 3,7-dirhamnoside (Kaempferitrin)	1.82E+04	2.17E+04	6.86E-01	1.19E+00	2.56E-01
pme0361	Quercetin 3-alpha-L-arabinofuranoside (Avicularin)	3.09E+04	3.73E+04	6.67E-01	1.21E+00	2.70E-01
pme3137	3-Hydroxyflavone	1.47E+04	1.54E+04	6.54E-01	1.05E+00	7.04E-02
pma0787	Quercetin-3-(6''-malonyl)-Glucoside	1.69E+04	1.60E+04	5.52E-01	9.45E-01	-8.15E-02
pme0369	Kaempferol 3-O-rutinoside (Nicotiflorin)	1.12E+05	1.01E+05	5.29E-01	9.06E-01	-1.43E-01
pme1539	Isorhamnetin 3-O-neohesperidoside	9.29E+03	7.67E+03	4.39E-01	8.25E-01	-2.78E-01
pme2977	Troxerutin (Trihydroxyethyl rutin)	9.32E+05	9.60E+05	2.70E-01	1.03E+00	4.24E-02

pmb0565	Syringetin 3-O-hexoside	3.43E+04	3.37E+04	2.26E-01	9.83E-01	-2.54E-02
pmb3013	Isorhamnetin O-acetyl-hexoside	2.47E+04	2.58E+04	1.98E-01	1.04E+00	5.90E-02
pme3396	Fustin	2.32E+04	2.33E+04	1.64E-01	1.00E+00	5.79E-03
pme1605	Kaempferol 3-O-robinobioside (Biorobin)	9.40E+04	9.35E+04	8.66E-02	9.95E-01	-7.49E-03
**Indole derivatives**						
pme2720	Indole-3-carboxaldehyde	5.46E+04	4.19E+04	1.59E+00	7.69E-01	-3.80E-01
pmb0818	Methoxyindoleacetic acid	1.11E+06	1.40E+06	1.44E+00	1.25E+00	3.27E-01
pmb1096	Indole	8.57E+04	9.29E+04	1.33E+00	1.08E+00	1.16E-01
pme0543	Indole-5-carboxylic acid	2.74E+05	2.17E+05	1.13E+00	7.89E-01	-3.42E-01
pme2607	5-Hydroxyindole-3-acetic acid	8.82E+05	7.29E+05	1.08E+00	8.26E-01	-2.75E-01
pme2836	5-Hydroxytryptophol	6.96E+05	5.80E+05	1.07E+00	8.33E-01	-2.64E-01
pmb0819	3-Indoleacetonitrile	3.75E+05	4.03E+05	9.73E-01	1.08E+00	1.04E-01
pmb0812	Imidazole-4-acetate	2.98E+05	2.91E+05	2.35E-01	9.78E-01	-3.21E-02
**Isoflavone**						
pme3251	Glycitein	1.49E+04	8.79E+03	1.59E+00	5.89E-01	-7.64E-01
pme3230	Calycosin	1.56E+04	9.97E+03	1.56E+00	6.41E-01	-6.41E-01
pme3276	2'-Hydroxygenistein	3.50E+03	1.30E+04	1.51E+00	3.70E+00	1.89E+00
pme3400	Sissotrin	1.64E+04	7.01E+04	1.46E+00	4.29E+00	2.10E+00
pme3292	Prunetin	6.50E+04	1.43E+05	1.39E+00	2.20E+00	1.14E+00

pme3210	Genistein 7-O-Glucoside (Genistin)	1.17E+05	2.21E+05	1.38E+00	1.89E+00	9.16E-01
pme1568	Orobol (5,7,3',4'-tetrahydroxyisoflavone)	7.90E+03	1.04E+04	1.26E+00	1.32E+00	3.96E-01
**Lipids**						
pma3606	9-Hydroxy-(10E,12Z,15Z)-octadecatrienoic acid	1.61E+06	1.16E+06	1.62E+00	7.19E-01	-4.75E-01
pmb2804	13-HPODE	1.06E+05	7.12E+04	1.60E+00	6.72E-01	-5.73E-01
pmb2778	9,10-EODE	3.51E+06	2.59E+06	1.59E+00	7.39E-01	-4.37E-01
pmb0889	Punicic acid	2.49E+06	1.85E+06	1.58E+00	7.45E-01	-4.25E-01
pmb2799	12,13-EODE	2.98E+05	2.60E+05	1.52E+00	8.72E-01	-1.98E-01
pmb2786	9-HOTrE	1.65E+06	1.23E+06	1.47E+00	7.46E-01	-4.23E-01
pmb0865	LysoPC 18:3 (2n isomer)	1.08E+06	2.56E+06	1.46E+00	2.36E+00	1.24E+00
pmb0161	DGMG (18:2) isomer1	2.60E+05	1.73E+05	1.44E+00	6.66E-01	-5.87E-01
pmb2383	MGMG (18:2) isomer2	5.53E+03	3.09E+03	1.39E+00	5.60E-01	-8.37E-01
pmb0863	LysoPC 16:2 (2n isomer)	1.39E+04	2.06E+04	1.38E+00	1.49E+00	5.71E-01
pmb1650	Octadeca-11E,13E,15Z-trienoic acid	1.22E+07	8.40E+06	1.36E+00	6.87E-01	-5.41E-01
pmb1574	Octadecadien-6-ynoic acid	3.02E+04	2.35E+04	1.35E+00	7.77E-01	-3.64E-01
pmb1562	MAG (18:4) isomer3	2.83E+04	2.45E+04	1.33E+00	8.66E-01	-2.07E-01
pmb1626	MGMG (18:2) isomer1	2.42E+04	1.43E+04	1.32E+00	5.93E-01	-7.55E-01
pmb2221	4-Hydroxysphinganine	1.74E+07	2.02E+07	1.31E+00	1.16E+00	2.18E-01
pmc0960	LysoPC 20:4	6.87E+06	5.69E+06	1.28E+00	8.28E-01	-2.72E-01

pmb0852	LysoPC 18:2	6.17E+06	5.26E+06	1.27E+00	8.52E-01	-2.31E-01
pmb0877	LysoPE 18:1	2.87E+04	2.23E+04	1.24E+00	7.78E-01	-3.62E-01
pmb0302	2-Aminoethylphosphonate	5.04E+04	6.24E+04	1.23E+00	1.24E+00	3.08E-01
pmb0168	PC 16:1/14:1	1.82E+04	5.39E+04	1.23E+00	2.97E+00	1.57E+00
pmf0294	1-Octadecanol	4.27E+04	4.04E+04	1.22E+00	9.47E-01	-7.81E-02
pmb0159	DGMG (18:2) isomer2	4.17E+04	2.43E+04	1.22E+00	5.84E-01	-7.77E-01
pmb2791	9-HpOTrE	3.59E+04	2.66E+04	1.20E+00	7.41E-01	-4.32E-01
pmb0856	LysoPE 18:1 (2n isomer)	7.19E+06	5.53E+06	1.20E+00	7.70E-01	-3.77E-01
pmb0882	LysoPC 18:1	5.56E+06	4.87E+06	1.10E+00	8.76E-01	-1.90E-01
pmb2251	DGMG (18:1)	1.06E+05	7.07E+04	1.10E+00	6.66E-01	-5.87E-01
pmb1605	MAG (18:3) isomer3	1.35E+05	9.81E+04	1.10E+00	7.25E-01	-4.64E-01
pmb2363	MAG (18:1) isomer1	9.30E+05	4.91E+05	1.08E+00	5.28E-01	-9.20E-01
pmb0160	MAG (18:3) isomer5	1.30E+06	7.97E+05	1.08E+00	6.11E-01	-7.11E-01
pmb2388	LysoPC 18:0 (2n isomer)	6.98E+05	6.07E+05	1.06E+00	8.69E-01	-2.02E-01
pmb0890	MAG (18:2)	9.17E+05	7.00E+05	1.05E+00	7.63E-01	-3.90E-01
pmb0163	DGMG (18:2) isomer3	8.54E+04	6.78E+04	1.05E+00	7.94E-01	-3.33E-01
pmb2789	13-HpOTrE(r)	3.30E+04	2.81E+04	1.03E+00	8.51E-01	-2.32E-01
pma0461	14,15-Dehydrocrepenynic acid	1.10E+06	5.96E+05	1.03E+00	5.39E-01	-8.90E-01
pmb2640	Lauric acid (C12:0)	2.87E+04	2.57E+04	1.02E+00	8.95E-01	-1.60E-01

pmb0128	delta-Tridecalactone	4.80E+04	4.38E+04	1.01E+00	9.14E-01	-1.30E-01
pmb5304	α-Linolenic acid	1.47E+04	2.04E+04	9.92E-01	1.39E+00	4.76E-01
pmb0874	LysoPE 18:2 (2n isomer)	1.65E+06	1.43E+06	9.85E-01	8.69E-01	-2.03E-01
pmb0848	LysoPC 16:1 (2n isomer)	8.42E+05	9.46E+05	9.74E-01	1.12E+00	1.67E-01
pmf0297	1-Eicosanol	1.96E+04	2.53E+04	9.29E-01	1.29E+00	3.70E-01
pmb2792	13-HOTrE(r)	3.03E+05	2.17E+05	9.24E-01	7.14E-01	-4.87E-01
pmb1656	MAG (18:3) isomer4	2.28E+05	2.67E+05	9.22E-01	1.17E+00	2.26E-01
pmb0165	LysoPC 16:1	2.58E+04	3.85E+04	8.70E-01	1.49E+00	5.79E-01
pmb2444	MAG (18:3) isomer1	7.61E+05	9.39E+05	8.67E-01	1.23E+00	3.04E-01
pmb2787	9-KODE	1.99E+05	2.32E+05	8.45E-01	1.17E+00	2.20E-01
pmb0876	LysoPE 16:0	1.43E+07	1.68E+07	8.14E-01	1.18E+00	2.33E-01
pmb2260	LysoPC 15:1	5.50E+04	4.92E+04	8.01E-01	8.94E-01	-1.62E-01
pmd0132	LysoPC 16:0 (2n isomer)	1.25E+07	1.47E+07	7.42E-01	1.18E+00	2.33E-01
pmb0880	LysoPE 18:0 (2n isomer)	3.93E+03	3.13E+03	7.31E-01	7.96E-01	-3.29E-01
pmb0885	4-oxo-9Z,11Z,13E,15E-octadecatetraenoic acid	7.77E+04	7.25E+04	7.28E-01	9.34E-01	-9.92E-02
pmb0855	LysoPC 16:0	1.05E+07	1.22E+07	6.90E-01	1.17E+00	2.28E-01
pmb0883	LysoPE 18:0	5.71E+04	5.21E+04	6.69E-01	9.13E-01	-1.31E-01
pmb0873	LysoPC 18:2 (2n isomer)	2.53E+06	2.76E+06	6.37E-01	1.09E+00	1.26E-01
pmb0859	LysoPC 18:1 (2n isomer)	4.14E+06	3.83E+06	6.29E-01	9.26E-01	-1.11E-01

pmb0854	LysoPC 18:3	1.60E+07	1.73E+07	6.10E-01	1.08E+00	1.14E-01
pmd0130	LysoPC 14:0 (2n isomer)	3.45E+05	3.27E+05	6.01E-01	9.46E-01	-8.05E-02
pmb2369	PC 19:2/16:0	4.02E+04	5.10E+04	5.93E-01	1.27E+00	3.40E-01
pma1303	LysoPC 16:2	5.88E+05	6.96E+05	5.79E-01	1.18E+00	2.42E-01
pmf0399	cis-Gondoic acid	1.42E+06	1.31E+06	5.69E-01	9.26E-01	-1.11E-01
pmb2228	LysoPC 19:0	2.55E+04	2.28E+04	5.65E-01	8.91E-01	-1.66E-01
pmf0396	Linoleic acid	1.18E+04	1.14E+04	5.48E-01	9.67E-01	-4.85E-02
pme2827	Palmitaldehyde	2.59E+07	2.79E+07	5.19E-01	1.08E+00	1.11E-01
pmb0866	LysoPC 14:0	1.11E+05	1.05E+05	5.18E-01	9.48E-01	-7.67E-02
pmb0296	MAG (18:1) isomer2	1.57E+05	1.41E+05	4.95E-01	9.01E-01	-1.51E-01
pmf0402	Lignoceric acid	7.78E+04	6.61E+04	4.31E-01	8.50E-01	-2.35E-01
pmb2406	LysoPC 17:0	4.92E+05	5.30E+05	4.15E-01	1.08E+00	1.07E-01
pmd0158	LysoPE 14:0 (2n isomer)	2.33E+03	2.16E+03	4.03E-01	9.26E-01	-1.10E-01
pmb0292	Cholesterol	2.63E+05	2.72E+05	3.92E-01	1.04E+00	5.16E-02
pmd0136	LysoPC 18:0	4.93E+06	5.34E+06	3.18E-01	1.08E+00	1.13E-01
pmb2643	Myristoleic acid (C14:1)	4.99E+03	4.70E+03	3.17E-01	9.41E-01	-8.74E-02
pmd0160	LysoPE 16:0 (2n isomer)	8.45E+05	8.89E+05	2.81E-01	1.05E+00	7.40E-02
pmd0145	LysoPC 20:1	1.05E+06	1.11E+06	2.03E-01	1.06E+00	8.55E-02
pmb2319	LysoPC 15:0	2.36E+06	2.33E+06	1.98E-01	9.88E-01	-1.68E-02

pmb0864	LysoPE 14:0	1.48E+05	1.47E+05	1.38E-01	9.95E-01	-7.92E-03
pmd0144	LysoPC 20:1 (2n isomer)	8.15E+05	8.58E+05	1.00E-01	1.05E+00	7.28E-02
pmb0164	MAG (18:2) isomer1	3.87E+03	3.70E+03	8.86E-02	9.55E-01	-6.61E-02
**Nucleotide and derivates**						
pme1363	Nicotinic acid adenine dinucleotide	5.06E+05	3.19E+05	1.60E+00	6.31E-01	-6.64E-01
pme1109	Guanine	1.10E+06	6.91E+05	1.59E+00	6.29E-01	-6.70E-01
pme3732	Cytidine	3.07E+06	1.30E+06	1.57E+00	4.23E-01	-1.24E+00
pmd0023	Adenosine	2.00E+06	1.63E+06	1.47E+00	8.14E-01	-2.97E-01
pmb0202	Xanthine	1.62E+05	2.72E+05	1.46E+00	1.67E+00	7.43E-01
pme0183	2-Hydroxy-6-aminopurine	2.69E+05	1.86E+05	1.43E+00	6.90E-01	-5.36E-01
pme0040	Adenine	8.86E+06	6.74E+06	1.42E+00	7.61E-01	-3.94E-01
pme3983	Hypoxanthine-9-β-D-arabinofuranoside	2.03E+06	4.11E+06	1.34E+00	2.02E+00	1.02E+00
pme3184	2'-Deoxyadenosine-5'-monophosphate	3.29E+05	3.82E+05	1.30E+00	1.16E+00	2.14E-01
pme1473	5'-Deoxy-5'-(methylthio)adenosine	5.94E+05	5.31E+05	1.28E+00	8.94E-01	-1.62E-01
pme0108	5-Methylcytosine	2.23E+04	1.35E+04	1.25E+00	6.04E-01	-7.27E-01
pmc0304	Succinyladenosine	6.91E+05	7.98E+05	1.24E+00	1.15E+00	2.06E-01
pme1119	Inosine	6.34E+05	4.54E+05	1.21E+00	7.16E-01	-4.81E-01
pme1184	Deoxyguanosine	1.39E+05	1.23E+05	1.21E+00	8.82E-01	-1.81E-01
pmb0514	Adenosine 3'-monophosphate	8.17E+05	1.15E+06	1.19E+00	1.41E+00	4.93E-01

pme3007	Uridine 5’-diphosphate	2.12E+05	2.53E+05	1.16E+00	1.19E+00	2.56E-01
pmb2922	Uridine 5'-diphospho-D-glucose	6.11E+06	4.45E+06	1.15E+00	7.28E-01	-4.58E-01
pme0264	Thymidine	3.51E+05	2.69E+05	1.15E+00	7.64E-01	-3.88E-01
pme3961	Deoxyadenosine	1.61E+06	1.47E+06	1.13E+00	9.10E-01	-1.37E-01
pme2746	Flavin adenine dinucleotide (FAD)	3.82E+05	3.60E+05	1.13E+00	9.42E-01	-8.63E-02
pmc0066	2'-Deoxyinosine-5'-monophosphate	4.65E+04	4.00E+04	1.12E+00	8.61E-01	-2.16E-01
pme3967	2-(dimethylamino)guanosine	3.07E+05	2.97E+05	1.07E+00	9.66E-01	-5.03E-02
pmb0532	Inosine 5'-monophosphate	8.47E+04	1.14E+05	1.05E+00	1.35E+00	4.33E-01
pme3336	N6-Succinyl Adenosine	1.67E+05	1.87E+05	1.03E+00	1.12E+00	1.64E-01
pme0033	Hypoxanthine	3.91E+04	4.73E+04	1.02E+00	1.21E+00	2.74E-01
pmb0998	Guanosine 5'-monophosphate	4.83E+04	6.20E+04	1.02E+00	1.28E+00	3.60E-01
pmf0118	Uridine 5'-diphosphoglucose disodium salt	5.94E+06	4.62E+06	9.73E-01	7.79E-01	-3.61E-01
pmc0274	6-Methylmercaptopurine	4.78E+06	5.44E+06	9.67E-01	1.14E+00	1.87E-01
pmf0136	UDP-α-D-glucose	7.17E+06	5.56E+06	9.16E-01	7.76E-01	-3.65E-01
pme1296	Xanthosine	7.71E+06	9.31E+06	9.14E-01	1.21E+00	2.73E-01
pmf0289	Riboprine	8.92E+03	7.34E+03	9.04E-01	8.23E-01	-2.81E-01
pme1187	5-Methyluridine	3.39E+03	4.52E+03	8.11E-01	1.33E+00	4.14E-01
pme1346	8-Hydroxyguanosine	3.18E+04	2.69E+04	7.86E-01	8.46E-01	-2.41E-01
pme3104	1-Methyladenine	1.15E+05	9.96E+04	7.81E-01	8.69E-01	-2.03E-01

pme3970	β-Pseudouridine	3.19E+04	2.69E+04	7.71E-01	8.43E-01	-2.46E-01
pme3197	Cyclic AMP	3.22E+05	3.84E+05	7.58E-01	1.19E+00	2.51E-01
pme3174	Cytidine 5'-monophosphate (Cytidylic acid)	2.04E+04	1.59E+04	7.10E-01	7.81E-01	-3.57E-01
pme0258	Uracil	3.28E+05	2.84E+05	7.09E-01	8.66E-01	-2.08E-01
pme1194	Deoxycytidine	1.54E+04	1.32E+04	7.05E-01	8.55E-01	-2.25E-01
pme2776	2'-Deoxyinosine	9.83E+04	9.35E+04	6.89E-01	9.51E-01	-7.18E-02
pmf0287	3'-dephospho-CoA	7.60E+04	8.25E+04	6.89E-01	1.09E+00	1.19E-01
pme1378	1-Methyladenosine	1.16E+04	1.43E+04	6.85E-01	1.24E+00	3.06E-01
pme0038	Cytosine	5.48E+03	6.89E+03	5.89E-01	1.26E+00	3.29E-01
pme2651	NADP	4.74E+05	4.39E+05	5.73E-01	9.26E-01	-1.11E-01
pme2801	7-Methylxanthine	1.07E+04	1.16E+04	5.38E-01	1.09E+00	1.22E-01
pme3200	1-methylguanidine	9.51E+04	8.82E+04	5.36E-01	9.27E-01	-1.09E-01
pme1175	Guanosine	1.92E+06	2.11E+06	5.35E-01	1.10E+00	1.39E-01
pmc0281	Adenosine O-ribose	6.58E+05	6.40E+05	5.33E-01	9.74E-01	-3.79E-02
pme2282	Guanosine monophosphate	1.19E+04	1.04E+04	5.28E-01	8.74E-01	-1.94E-01
pme3188	Uridine 5'-monophosphate	2.15E+04	2.36E+04	4.21E-01	1.10E+00	1.37E-01
pmb0197	N2-methylguanosine	1.98E+04	2.15E+04	4.11E-01	1.09E+00	1.19E-01
pme2555	Adenosine 5'-monophosphate	5.28E+04	5.40E+04	1.92E-01	1.02E+00	3.32E-02
pme1373	2'-Deoxycytidine-5'-monophosphate	2.78E+04	2.80E+04	1.67E-01	1.01E+00	1.31E-02

pme3838	Guanosine 3',5'-cyclic monophosphate	2.68E+05	2.73E+05	1.43E-01	1.02E+00	3.03E-02
pmf0596	Cordycepin	2.43E+04	2.41E+04	6.16E-02	9.89E-01	-1.55E-02
pme0028	Uridine	9.74E+06	9.73E+06	5.07E-02	9.99E-01	-1.79E-03
pme0152	5,6-Dihydro-5-methyluracil	6.71E+03	7.33E+03	2.71E-02	1.09E+00	1.27E-01
**Organic acids and derivatives**						
pme2241	Citraconic acid	5.77E+05	4.31E+05	1.53E+00	7.46E-01	-4.22E-01
pme2887	Guanidinoethyl sulfonate	5.50E+04	6.45E+04	1.51E+00	1.17E+00	2.31E-01
pme0233	Kynurenic acid	2.37E+05	2.08E+05	1.51E+00	8.78E-01	-1.87E-01
pme2050	Citric acid	5.53E+06	6.75E+06	1.50E+00	1.22E+00	2.88E-01
pmf0482	Cynarin	4.90E+05	6.84E+05	1.49E+00	1.40E+00	4.83E-01
pme2049	2-Hydroxybutanoic acid	3.35E+04	5.67E+04	1.48E+00	1.69E+00	7.59E-01
pmf0131	Cryptochlorogenic acid	2.00E+05	2.72E+05	1.46E+00	1.36E+00	4.43E-01
pme3719	D-Xylonic acid	9.10E+06	1.05E+07	1.46E+00	1.16E+00	2.10E-01
pme1816	Neochlorogenic acid (5-O-Caffeoylquinic acid)	5.43E+05	7.16E+05	1.46E+00	1.32E+00	3.99E-01
pmf0218	Phosphoenolpyruvate trisodium salt	7.34E+04	4.08E+04	1.46E+00	5.56E-01	-8.47E-01
pma3724	1-O-Feruloyl quinic acid	1.44E+04	2.11E+04	1.45E+00	1.46E+00	5.50E-01
pme3011	γ-aminobutyric acid	1.85E+06	2.36E+06	1.44E+00	1.27E+00	3.48E-01
pmb1912	10-Formyl-THF	2.54E+06	2.41E+06	1.44E+00	9.49E-01	-7.48E-02
pme3186	Dl-Glyceraldehyde3-phosphate	5.80E+05	8.04E+05	1.44E+00	1.39E+00	4.70E-01

pmb2928	Gallic acid O-Hexoside	1.13E+06	1.27E+06	1.43E+00	1.13E+00	1.70E-01
pmf0280	D-Galacturonic acid	7.65E+06	1.22E+07	1.41E+00	1.59E+00	6.72E-01
pmf0578	Citric acid monohydrate	3.74E+06	4.32E+06	1.41E+00	1.15E+00	2.07E-01
pme2706	2,3-Dihydroxybenzoic acid	9.30E+05	1.74E+06	1.41E+00	1.87E+00	9.04E-01
pme0085	Rosmarinic acid	3.04E+05	3.69E+05	1.40E+00	1.22E+00	2.81E-01
pme0399	Chlorogenic acid (3-O-Caffeoylquinic acid)	4.57E+06	6.41E+06	1.37E+00	1.40E+00	4.90E-01
pmf0424	Isochlorogenic acid B	7.36E+04	1.68E+05	1.37E+00	2.28E+00	1.19E+00
pme2541	α-Hydroxyisobutyric acid	1.80E+05	8.28E+05	1.37E+00	4.61E+00	2.20E+00
pme2761	4-Hydroxy-2-oxoglutaric acid	5.10E+05	6.99E+05	1.35E+00	1.37E+00	4.56E-01
pme2589	Oxoadipic acid	6.93E+04	5.75E+04	1.34E+00	8.29E-01	-2.70E-01
pme0272	Benzoylformic acid	1.87E+04	1.31E+04	1.34E+00	7.04E-01	-5.07E-01
pme2903	4-Hydroxybenzoic acid	2.46E+06	1.99E+06	1.34E+00	8.09E-01	-3.06E-01
pme0291	Dl-2-Aminooctanoic acid	8.88E+04	7.61E+04	1.33E+00	8.57E-01	-2.23E-01
pme0237	Ethyl 3,4-Dihydroxybenzoate (Ethyl protocatechuate)	5.84E+04	7.25E+04	1.29E+00	1.24E+00	3.12E-01
pme0275	4-Oxopentanoate	1.35E+04	1.86E+04	1.28E+00	1.37E+00	4.59E-01
pme0207	3-Hydroxybutyrate	3.93E+06	4.23E+06	1.25E+00	1.08E+00	1.04E-01
pme0486	Methylmalonic acid	4.50E+06	7.05E+06	1.24E+00	1.56E+00	6.46E-01
pme3083	2-(Formylamino)benzoic acid	1.99E+05	1.61E+05	1.23E+00	8.10E-01	-3.05E-01
pme1830	Succinic acid	4.75E+06	7.15E+06	1.22E+00	1.50E+00	5.88E-01

pmb1318	8-Methyl-2-oxo-4-phenyl-2H-chromen-7-yl 4-(hexyloxy)benzoate	5.46E+05	4.19E+05	1.19E+00	7.68E-01	-3.81E-01
pme1683	D-Pantothenic dcid	1.39E+04	2.35E+04	1.19E+00	1.69E+00	7.58E-01
pme2033	L(-)-Malic acid	3.88E+06	3.05E+06	1.17E+00	7.87E-01	-3.46E-01
pmb2556	Syringaldehyde O-glucoside	1.19E+04	9.18E+03	1.16E+00	7.73E-01	-3.72E-01
pme2169	Fumaric acid	2.57E+06	2.24E+06	1.16E+00	8.72E-01	-1.98E-01
pme2601	3-Hydroxypropanoic acid	2.91E+05	3.36E+05	1.16E+00	1.16E+00	2.09E-01
pme2129	(S)-(-)-2-Hydroxyisocaproic acid	2.59E+04	7.88E+04	1.15E+00	3.05E+00	1.61E+00
pme1806	Chlorogenic acid methyl ester	2.26E+04	4.39E+04	1.14E+00	1.94E+00	9.56E-01
pme1711	3-Hydroxy-3-methyl butyric acid	7.18E+05	4.48E+05	1.14E+00	6.24E-01	-6.80E-01
pme2709	2-Hydroxy-2-methyl butyric acid	6.25E+04	8.59E+04	1.13E+00	1.37E+00	4.58E-01
pme0271	Maleic acid	4.48E+04	3.04E+04	1.12E+00	6.79E-01	-5.58E-01
pmb3055	p-Coumaroyl quinic acid O-glucuronic acid	1.53E+04	2.24E+04	1.11E+00	1.47E+00	5.54E-01
pme0413	Vanillin	2.47E+06	2.15E+06	1.09E+00	8.71E-01	-1.99E-01
pmb2938	4-O-Caffeoyl quinic acid (criptochlorogenic acid)	1.18E+05	1.06E+05	1.09E+00	9.00E-01	-1.53E-01
pmf0420	Benzyl acetate	8.58E+04	9.51E+04	1.09E+00	1.11E+00	1.49E-01
pme2380	A-Ketoglutaric acid	3.69E+06	3.52E+06	1.08E+00	9.54E-01	-6.75E-02
pme1814	2,5-dihydroxybenzoic acid (Gentisic acid)	5.05E+05	6.44E+05	1.06E+00	1.28E+00	3.51E-01
pme3034	ethylmalonate	8.65E+04	7.05E+04	1.04E+00	8.15E-01	-2.95E-01
pme3096	Aminomalonic acid	3.64E+06	5.00E+06	1.04E+00	1.37E+00	4.59E-01

pme0245	Adipic acid	5.10E+05	3.86E+05	1.01E+00	7.57E-01	-4.01E-01
pme0295	4-Acetamidobutyric acid	1.17E+05	1.80E+05	1.01E+00	1.55E+00	6.28E-01
pme0286	2-Hydroxyisocaproic acid	2.84E+04	9.41E+04	9.81E-01	3.32E+00	1.73E+00
pmb0247	p-Aminobenzoate	6.91E+05	5.69E+05	9.74E-01	8.24E-01	-2.80E-01
pmf0413	Propyl gallate	3.41E+04	5.55E+04	9.60E-01	1.63E+00	7.05E-01
pmb2826	Citramalate	1.64E+07	1.35E+07	9.53E-01	8.25E-01	-2.78E-01
pme3009	trans-Citridic acid	1.46E+06	1.32E+06	9.20E-01	9.06E-01	-1.42E-01
pme3309	2-Methylglutaric acid	4.88E+05	3.84E+05	9.09E-01	7.87E-01	-3.45E-01
pmb0751	5-O-p-Coumaroyl shikimic acid	1.81E+04	2.25E+04	9.04E-01	1.24E+00	3.16E-01
pmf0245	Kojic acid	1.17E+05	1.74E+05	8.98E-01	1.48E+00	5.70E-01
pmb3059	Quinic acid O-di-glucuronic acid	1.59E+04	1.90E+04	8.93E-01	1.19E+00	2.55E-01
pme0309	Methyl gallate	1.41E+05	2.95E+05	8.83E-01	2.09E+00	1.07E+00
pmb3068	1-O-p-Coumaroyl quinic acid	1.36E+04	1.16E+04	8.82E-01	8.54E-01	-2.28E-01
pme1977	Suberic acid	3.28E+05	2.57E+05	8.69E-01	7.83E-01	-3.53E-01
pmb3074	3-O-p-Coumaroyl quinic acid	6.47E+04	8.17E+04	8.28E-01	1.26E+00	3.35E-01
pme0281	Terephthalic acid	1.99E+07	1.84E+07	8.24E-01	9.26E-01	-1.11E-01
pme0267	2-Methylsuccinic acid	7.83E+06	7.12E+06	7.96E-01	9.09E-01	-1.38E-01
pme0243	Glutaric acid	6.71E+05	5.77E+05	7.91E-01	8.60E-01	-2.18E-01
pme0241	Benzoic acid	1.27E+05	6.21E+04	7.91E-01	4.88E-01	-1.03E+00

pme0395	2-Methoxybenzoic acid	1.37E+04	7.59E+03	7.91E-01	5.56E-01	-8.46E-01
pmb3072	3-O-p-coumaroyl shikimic acid O-hexoside	4.89E+04	4.14E+04	7.86E-01	8.47E-01	-2.40E-01
pme1852	Creatine	1.75E+04	2.43E+04	7.69E-01	1.39E+00	4.71E-01
pma2681	Xanthurenic acid	3.67E+04	3.13E+04	7.58E-01	8.53E-01	-2.30E-01
pme0250	Azelaic acid	8.80E+05	7.36E+05	7.56E-01	8.37E-01	-2.56E-01
pmb2657	Argininosuccinate	2.08E+06	2.39E+06	7.53E-01	1.15E+00	2.03E-01
pme0265	Sebacate	1.94E+04	2.33E+04	6.98E-01	1.20E+00	2.62E-01
pme0274	6-Aminocaproic acid	2.65E+07	2.98E+07	6.71E-01	1.12E+00	1.70E-01
pme2603	3-Hydroxyanthranilic acid	4.71E+04	5.63E+04	6.52E-01	1.20E+00	2.58E-01
pmb0829	Phosphoric acid	2.89E+06	2.71E+06	6.48E-01	9.38E-01	-9.26E-02
pme2036	Quinic acid	5.68E+06	6.12E+06	6.07E-01	1.08E+00	1.08E-01
pme3154	(Rs)-Mevalonic acid	3.16E+05	2.81E+05	6.06E-01	8.89E-01	-1.70E-01
pmb3101	2-Isopropylmalate	2.28E+07	2.19E+07	6.00E-01	9.59E-01	-6.02E-02
pme2671	4-Hydroxybenzaldehyde	5.17E+05	4.89E+05	5.75E-01	9.47E-01	-7.85E-02
pme1218	2-Picolinic acid	9.84E+03	1.14E+04	5.38E-01	1.16E+00	2.17E-01
pmb0752	3-O-Feruloyl quinic acid	1.98E+04	2.39E+04	5.17E-01	1.21E+00	2.73E-01
pme0289	4-Ethylbenzoic acid	1.06E+04	9.27E+03	5.09E-01	8.75E-01	-1.92E-01
pme0284	4-Guanidinobutyric acid	2.27E+04	2.03E+04	5.05E-01	8.93E-01	-1.64E-01
pmf0196	Phosphoenolpyruvic acid	9.22E+04	8.34E+04	4.93E-01	9.05E-01	-1.44E-01

pmb2554	5-O-Feruloyl quinic acid glucoside	1.45E+04	1.57E+04	4.82E-01	1.09E+00	1.18E-01
pmb3066	5-O-p-coumaroyl shikimic acid O-hexoside	2.96E+05	2.77E+05	4.55E-01	9.36E-01	-9.56E-02
pme0283	Phthalic acid	1.23E+07	1.18E+07	4.49E-01	9.56E-01	-6.48E-02
pmb1587	4-Hydroxy-3,5-diisopropylbenzaldehyde	9.65E+03	1.01E+04	4.44E-01	1.05E+00	7.27E-02
pme1936	Creatinine	1.79E+04	1.95E+04	3.97E-01	1.09E+00	1.25E-01
pme0397	Shikimic acid	4.82E+05	4.70E+05	3.76E-01	9.74E-01	-3.77E-02
pmb3107	Syringic acid O-glucoside	8.70E+04	8.40E+04	3.66E-01	9.66E-01	-4.93E-02
pma6281	Methylglutaric acid	1.71E+04	1.63E+04	3.43E-01	9.52E-01	-7.11E-02
pme2598	3,4-Dihydroxybenzeneacetic acid	3.47E+05	3.41E+05	2.95E-01	9.84E-01	-2.38E-02
pmb0766	Azoxystrobin acid	5.84E+05	6.16E+05	2.84E-01	1.05E+00	7.61E-02
pme0269	Dodecanedioic aicd	2.03E+04	1.97E+04	2.33E-01	9.74E-01	-3.85E-02
pmb2497	4-Hydroxy-3-methoxymandelate	9.87E+03	1.07E+04	2.29E-01	1.08E+00	1.17E-01
pmb2654	Anthranilate O-hexosyl-O-hexoside	1.75E+05	1.71E+05	1.92E-01	9.77E-01	-3.39E-02
pme3207	trans,trans-Muconic acid	1.65E+06	1.67E+06	1.86E-01	1.01E+00	1.68E-02
pme0049	2-Aminoethanesulfinic acid	1.82E+04	1.92E+04	1.52E-01	1.05E+00	7.04E-02
pmb2871	2,5-dihydroxy benzoic acid O-hexside	2.63E+06	2.69E+06	1.34E-01	1.02E+00	3.08E-02
pma3649	5-Aminolevulinate	2.65E+07	2.72E+07	1.05E-01	1.03E+00	3.99E-02
pmf0408	Plantamajoside	1.47E+04	1.53E+04	9.80E-02	1.04E+00	5.92E-02
pme2009	L-(+)-Tartaric acid	5.13E+04	5.20E+04	7.41E-02	1.01E+00	1.79E-02

pme3093	(S)-2-(4-Aminobutanamido)-3-(1-methyl-1H-imidazol-5-yl)propanoic acid	5.46E+03	5.22E+03	6.62E-02	9.56E-01	-6.45E-02
pme3069	Taurocholic acid	7.32E+06	7.36E+06	5.45E-02	1.01E+00	8.17E-03
pmf0425	D-tartaric acid	6.21E+04	6.22E+04	5.30E-02	1.00E+00	7.74E-04
pmf0419	Salicin	7.23E+04	7.23E+04	3.01E-02	1.00E+00	-1.33E-04
pma0675	o-Anisic acid	1.45E+04	1.50E+04	6.75E-03	1.04E+00	5.57E-02
pmb3099	Diethyl phosphate	2.35E+07	2.32E+07	3.47E-03	9.87E-01	-1.86E-02
**Others**						
pmf0534	Hinokitiol	4.96E+04	2.78E+04	1.56E+00	5.60E-01	-8.36E-01
pmb3079	N-Acetylglucosamine 1-phosphate	1.26E+05	1.06E+05	1.53E+00	8.43E-01	-2.47E-01
pma2400	Dihydrojasmone	4.74E+04	5.75E+04	1.50E+00	1.21E+00	2.76E-01
pma0948	Hydroxyphenethylamine	5.76E+04	3.01E+05	1.50E+00	5.22E+00	2.38E+00
pmb0374	Aminopurine	1.72E+06	1.50E+06	1.46E+00	8.72E-01	-1.98E-01
pmf0571	6-Aminopenicillanic acid	1.45E+04	7.53E+03	1.40E+00	5.19E-01	-9.45E-01
pme2433	Diethanolamine	2.28E+04	2.72E+04	1.40E+00	1.19E+00	2.53E-01
pme2830	O-Phosphorylethanolamine	6.62E+04	5.98E+04	1.39E+00	9.03E-01	-1.47E-01
pmb1652	Phthalic anhydride	1.59E+06	1.10E+06	1.35E+00	6.95E-01	-5.26E-01
pmf0217	D-Glucose-6-phosphate disodium salt	4.78E+06	3.95E+06	1.23E+00	8.26E-01	-2.76E-01
pmf0261	Polydatin	6.42E+03	8.62E+03	1.16E+00	1.34E+00	4.26E-01

pme3705	D-glucoronic acid	5.48E+06	8.28E+06	1.11E+00	1.51E+00	5.96E-01
pmb0064	DIMBOA glucoside	5.19E+04	4.26E+04	1.05E+00	8.21E-01	-2.85E-01
pmb3081	Glucarate O-Phosphoric acid	4.06E+06	4.73E+06	8.95E-01	1.16E+00	2.20E-01
pmf0416	α-Ionone	1.55E+04	1.03E+04	8.94E-01	6.63E-01	-5.94E-01
pmf0516	Mangiferin	1.34E+04	1.15E+04	8.66E-01	8.54E-01	-2.28E-01
pmf0522	alpha-Santonin	1.50E+04	8.32E+03	8.04E-01	5.53E-01	-8.54E-01
pme0534	Gluconic acid	2.98E+06	3.48E+06	7.87E-01	1.17E+00	2.25E-01
pmf0606	Pectin (Technical Grade)	2.34E+05	1.62E+05	7.28E-01	6.93E-01	-5.28E-01
pmf0347	Dihydropinosylvin	1.33E+04	1.13E+04	7.19E-01	8.49E-01	-2.35E-01
pmf0519	Bergenin	1.67E+04	1.52E+04	6.90E-01	9.10E-01	-1.36E-01
pmb1240	Phellodensin F	1.91E+06	1.76E+06	4.65E-01	9.18E-01	-1.23E-01
pmf0230	Forchlorfenuron	3.49E+04	3.09E+04	4.44E-01	8.85E-01	-1.76E-01
pme2828	4-Nitrophenol	2.51E+07	2.59E+07	4.40E-01	1.03E+00	4.16E-02
pmb2507	2-Deoxyribose 1-phosphate	1.52E+06	1.42E+06	4.32E-01	9.33E-01	-9.95E-02
pmf0243	Benzyl β-D-Glucopyranoside	6.69E+04	6.56E+04	4.19E-01	9.79E-01	-3.00E-02
pme2255	L-Gulonic-γ-lactone	2.53E+04	2.26E+04	3.76E-01	8.95E-01	-1.61E-01
pmf0518	Dehydrocostus lactone	6.39E+03	6.01E+03	3.52E-01	9.41E-01	-8.85E-02
pmb1452	N-Lauryldiethanolamine	1.73E+05	1.68E+05	2.48E-01	9.71E-01	-4.18E-02
pmf0141	2-Phenylethanol	7.69E+05	5.96E+05	2.23E-01	7.75E-01	-3.67E-01

pme2366	Phenethylamine	1.44E+06	1.42E+06	2.15E-01	9.89E-01	-1.62E-02
pmf0314	ABIENOL	9.36E+03	9.46E+03	1.18E-01	1.01E+00	1.63E-02
pmb0069	Benzamide	1.54E+06	1.56E+06	1.38E-02	1.01E+00	1.31E-02
**Phenolamides**						
pme2292	Putrescine	4.01E+06	4.70E+06	1.41E+00	1.17E+00	2.29E-01
pmb0490	N-p-Coumaroyl putrescine	2.42E+05	7.55E+05	1.31E+00	3.12E+00	1.64E+00
pmb0896	N'-Feruloyl putrescine	1.85E+04	4.90E+04	1.30E+00	2.65E+00	1.41E+00
pmb0505	N'-p-Coumaroyl putrescine	1.58E+05	4.86E+05	1.27E+00	3.08E+00	1.62E+00
pmb0508	N-p-Coumaroyl agmatine	2.96E+03	1.13E+04	1.23E+00	3.83E+00	1.94E+00
pmd0017	Spermine	2.50E+07	2.72E+07	1.22E+00	1.09E+00	1.18E-01
pmb0501	Agmatine	2.08E+05	2.73E+05	1.22E+00	1.31E+00	3.92E-01
pmb0907	N-Feruloyl putrescine	4.28E+06	5.98E+06	1.03E+00	1.40E+00	4.85E-01
pme2693	N-Acetylputrescine	2.03E+05	2.55E+05	9.98E-01	1.26E+00	3.33E-01
pma0702	N', N''-disinapoylspermidine	7.94E+06	7.47E+06	8.70E-01	9.41E-01	-8.77E-02
pmb0323	N-Caffeoyl putrescine	4.44E+03	1.18E+04	8.57E-01	2.66E+00	1.41E+00
pme1841	1,5-Diaminopentane	4.11E+04	4.70E+04	7.60E-01	1.15E+00	1.95E-01
pmb0492	"N',N"",N"""-p-coumaroyl-cinnamoyl-caffeoyl spermidine"	2.87E+04	2.62E+04	7.03E-01	9.12E-01	-1.33E-01
pmb0130	N-Acetyl tryptamine	2.45E+03	6.52E+03	6.25E-01	2.66E+00	1.41E+00
pmb0494	N-Sinapoyl putrescine	1.96E+05	2.04E+05	3.73E-01	1.04E+00	6.11E-02

pmb2893	N-p-Coumaroyl hydroxyagmatine	8.23E+03	8.09E+03	2.38E-01	9.82E-01	-2.61E-02
pma0170	N', N''-Diferuloylspermidine	1.32E+04	1.30E+04	1.36E-01	9.85E-01	-2.25E-02
**Phenylpropanoids**						
pme1640	sesamolin	5.72E+06	3.77E+06	1.56E+00	6.60E-01	-5.99E-01
pma6561	Caffeic acid O-glucoside	8.33E+05	1.37E+06	1.55E+00	1.65E+00	7.22E-01
pme0408	Syringic acid	2.32E+04	1.52E+04	1.55E+00	6.57E-01	-6.06E-01
pme3553	Psoralen	5.76E+03	1.76E+04	1.55E+00	3.05E+00	1.61E+00
pme2988	3,4-Dihydrocoumarin	4.61E+04	2.16E+04	1.53E+00	4.69E-01	-1.09E+00
pmf0284	4-Hydroxy-3-methoxycinnamaldehyde	2.67E+06	1.98E+06	1.51E+00	7.44E-01	-4.28E-01
pme0422	3-Hydroxy-4-methoxycinnamic acid	1.61E+05	1.22E+05	1.49E+00	7.63E-01	-3.90E-01
pmb3110	Vanillic acid	3.02E+05	2.40E+05	1.48E+00	7.96E-01	-3.28E-01
pmf0295	Skimmin	2.59E+06	1.89E+06	1.46E+00	7.30E-01	-4.54E-01
pme1646	Pinoresinol	4.79E+05	3.83E+05	1.44E+00	8.01E-01	-3.20E-01
pme0303	Caffeate	2.49E+05	2.00E+05	1.41E+00	8.04E-01	-3.14E-01
pmf0014	Scopolin	2.80E+06	2.14E+06	1.37E+00	7.63E-01	-3.90E-01
pmf0558	Angelicin	1.84E+03	1.35E+04	1.35E+00	7.32E+00	2.87E+00
pmb3093	6,7-dihydroxycoumarin 7-O-quinic acid	3.60E+04	4.36E+04	1.35E+00	1.21E+00	2.77E-01
pme0305	Ferulic acid	1.30E+05	1.15E+05	1.32E+00	8.84E-01	-1.77E-01
pmb2795	4-Methoxycinnamic acid	6.03E+03	7.30E+03	1.31E+00	1.21E+00	2.75E-01

pmf0094	Methyl p-coumarate	2.29E+03	1.05E+04	1.30E+00	4.59E+00	2.20E+00
pme0429	3,4,5-Trimethoxycinnamic acid	2.09E+04	4.35E+03	1.27E+00	2.08E-01	-2.26E+00
pmf0605	Notopterol	3.44E+04	2.57E+04	1.26E+00	7.47E-01	-4.21E-01
pme3416	Esculetin (6,7-dihydroxycoumarin)	4.29E+04	3.17E+04	1.25E+00	7.38E-01	-4.37E-01
pmf0591	Cinnamic acid	4.31E+04	1.54E+05	1.23E+00	3.57E+00	1.84E+00
pmf0498	Eleutheroside E	6.99E+03	9.82E+03	1.20E+00	1.41E+00	4.91E-01
pme0300	trans-Cinnamate	5.38E+04	1.63E+05	1.20E+00	3.03E+00	1.60E+00
pmf0608	Umbelliferone	5.10E+04	6.55E+04	1.20E+00	1.28E+00	3.62E-01
pmf0406	Phenethyl caffeate	9.00E+00	2.77E+03	1.20E+00	3.07E+02	8.26E+00
pmb0423	Hydroxy-methoxycinnamate	1.90E+05	3.05E+05	1.19E+00	1.61E+00	6.85E-01
pmf0271	(E)-p-coumaric acid	2.89E+05	2.36E+05	1.17E+00	8.14E-01	-2.96E-01
pme3453	p-Coumaraldehyde	1.72E+05	1.43E+05	1.16E+00	8.27E-01	-2.74E-01
pmf0067	Isoacteoside	8.83E+03	3.17E+03	1.16E+00	3.59E-01	-1.48E+00
pme0388	Homovanillic acid	4.55E+05	3.35E+05	1.13E+00	7.38E-01	-4.39E-01
pme1434	Syringin	4.50E+04	3.65E+04	1.13E+00	8.12E-01	-3.00E-01
pmb2940	1-O-beta-D-Glucopyranosyl sinapate	2.98E+05	3.63E+05	1.12E+00	1.22E+00	2.84E-01
pme3443	Sinapinaldehyde	3.44E+04	2.70E+04	1.10E+00	7.84E-01	-3.51E-01
pme1637	Coniferyl alcohol	6.69E+04	5.39E+04	1.09E+00	8.05E-01	-3.13E-01
pme3428	Esculin (6,7-Dihydroxycoumarin-6-glucoside)	1.01E+06	1.12E+06	1.06E+00	1.11E+00	1.48E-01

pme3305	p-Coumaryl alcohol	4.24E+05	3.89E+05	1.04E+00	9.18E-01	-1.23E-01
pmf0359	Skimmine	1.59E+05	1.24E+05	9.18E-01	7.84E-01	-3.51E-01
pmb4777	4-Hydroxy-7-methoxycoumarin-beta-rhamnoside	1.45E+04	1.30E+04	8.92E-01	8.98E-01	-1.56E-01
pmb0235	O-Feruloyl coumarin	1.11E+04	5.50E+03	8.22E-01	4.98E-01	-1.01E+00
pme0418	3-(4-Hydroxyphenyl)propionic acid	7.17E+03	1.17E+04	7.39E-01	1.64E+00	7.11E-01
pme3569	Scoparone	1.00E+04	9.41E+03	7.35E-01	9.38E-01	-9.25E-02
pmb0108	Feruloyl syringic acid	7.13E+04	8.27E+04	7.32E-01	1.16E+00	2.14E-01
pme2993	Scopoletin (7-Hydroxy-5-methoxycoumarin)	7.07E+04	9.07E+04	7.29E-01	1.28E+00	3.59E-01
pmf0098	trans-4-Hydroxycinnamic acid Methyl Ester	5.66E+03	6.55E+03	6.93E-01	1.16E+00	2.11E-01
pme1439	p-Coumaric acid	1.66E+05	1.52E+05	6.88E-01	9.15E-01	-1.28E-01
pmb0808	6,7-dihydroxycoumarin 6-O-quinic acid	1.84E+04	2.48E+04	6.72E-01	1.35E+00	4.33E-01
pmb3094	Esculetin O-quinacyl esculetin O-quinic acid	4.25E+03	5.73E+03	6.43E-01	1.35E+00	4.32E-01
pma6599	6-Hydroxymethylherniarin	1.02E+05	9.60E+04	5.95E-01	9.37E-01	-9.34E-02
pmf0440	4-Methoxycinna Maldehyde	2.80E+05	3.01E+05	5.61E-01	1.08E+00	1.05E-01
pme0307	Resveratrol	7.42E+03	8.86E+03	5.60E-01	1.19E+00	2.56E-01
pmb2933	Caftaric acid	3.89E+04	4.16E+04	5.54E-01	1.07E+00	9.90E-02
pmb0475	Gallic acid O-feruloyl-O-hexosyl-O-hexoside	2.26E+04	2.08E+04	5.39E-01	9.18E-01	-1.24E-01
pmb2620	3,4-Dimethoxycinnamic acid	4.75E+03	4.38E+03	5.00E-01	9.21E-01	-1.19E-01
pma0104	N-sinapoyl hydroxycoumarin	2.90E+04	5.47E+04	4.84E-01	1.89E+00	9.16E-01

pmf0319	Acetosyringone	2.11E+04	2.89E+04	4.48E-01	1.37E+00	4.50E-01
pmf0095	Methyl ferulate	4.16E+03	3.84E+03	4.18E-01	9.22E-01	-1.18E-01
pme3246	Coniferin	1.72E+07	1.74E+07	2.32E-01	1.01E+00	1.25E-02
pmb2835	Syringaldehyde	4.56E+04	4.40E+04	2.16E-01	9.65E-01	-5.10E-02
pmb0142	Caffeic aldehyde	3.62E+04	3.79E+04	2.08E-01	1.05E+00	6.73E-02
pmf0152	Brevifolincarboxylic acid	2.40E+05	2.39E+05	1.05E-01	9.96E-01	-6.02E-03
pme3425	6-Methoxy-7,8-DihydroxyCoumarin	3.65E+05	3.60E+05	9.33E-03	9.84E-01	-2.35E-02
**Polyphenol**						
pmf0458	6-Gingerol	1.31E+05	8.97E+04	1.61E+00	6.87E-01	-5.43E-01
pme0450	L-Epicatechin	2.63E+06	3.89E+06	1.37E+00	1.48E+00	5.63E-01
pme1516	Epigallocatechin (EGC)	7.89E+04	1.05E+05	1.20E+00	1.34E+00	4.18E-01
pme2482	Protocatechuic aldehyde	7.99E+04	9.32E+04	1.19E+00	1.17E+00	2.22E-01
pme1824	Protocatechuic acid	2.49E+06	3.24E+06	1.05E+00	1.30E+00	3.77E-01
pme0205	Catechin	1.42E+07	1.34E+07	9.45E-01	9.43E-01	-8.41E-02
pme1562	Epicatechin gallate (ECG)	1.46E+05	1.65E+05	9.10E-01	1.13E+00	1.72E-01
pme1535	(+)-Gallocatechin (GC)	1.96E+04	2.42E+04	8.89E-01	1.24E+00	3.05E-01
pmb0835	Gallocatechin-gallocatechin	4.60E+07	6.19E+07	5.85E-01	1.35E+00	4.28E-01
pmb2947	Catechin-catechin-catechin	6.67E+04	6.31E+04	4.48E-01	9.47E-01	-7.81E-02
pmb2831	Protocatechuic acid O-glucoside	4.34E+05	4.43E+05	2.99E-01	1.02E+00	3.17E-02

pme1488	Epigallate catechin gallate (EGCG)	1.37E+04	1.32E+04	2.69E-01	9.69E-01	-4.52E-02
pmb3114	Epicatechin-epiafzelechin	5.19E+04	5.13E+04	2.14E-01	9.88E-01	-1.72E-02
pme2247	Ellagic acid	4.34E+05	4.40E+05	1.93E-01	1.01E+00	1.83E-02
**Proanthocyanidins**						
pme0436	Procyanidin B3	1.67E+06	1.24E+06	1.23E+00	7.41E-01	-4.32E-01
pmb0837	Procyanidin A3	5.39E+04	3.66E+04	1.17E+00	6.79E-01	-5.58E-01
pme0435	Procyanidin B2	1.80E+05	2.97E+05	1.07E+00	1.65E+00	7.20E-01
pme0432	Procyanidin A2	1.24E+05	1.21E+05	1.02E-01	9.76E-01	-3.44E-02
pme0431	Procyanidin A1	1.11E+06	1.11E+06	2.05E-02	1.01E+00	1.02E-02
**Quinones**						
pmf0601	Purpurin	9.00E+00	4.23E+03	1.21E+00	4.70E+02	8.88E+00
pmf0541	Shikonin	6.00E+04	6.50E+04	1.08E+00	1.08E+00	1.16E-01
**Sterides**						
pmf0079	Soyasapogenol B	3.03E+05	2.84E+05	1.43E+00	9.35E-01	-9.64E-02
pmf0085	Cycloartenol	1.21E+05	1.14E+05	9.97E-01	9.46E-01	-8.00E-02
pmf0091	2,3-Oxidosqualene	1.28E+05	1.22E+05	7.86E-01	9.53E-01	-6.94E-02
pmf0070	β-Sitosterol	2.09E+07	1.97E+07	6.18E-01	9.46E-01	-8.02E-02
pmf0073	Campesterol	2.80E+03	2.74E+03	4.73E-01	9.80E-01	-2.99E-02
**Terpene**						

pmf0598	(+)-alpha-Pinene	1.34E+04	9.17E+03	1.67E+00	6.85E-01	-5.46E-01
pmf0286	Phytol	1.53E+06	1.12E+06	1.64E+00	7.32E-01	-4.50E-01
pmb1530	Phytocassane D	1.24E+04	1.15E+04	1.48E+00	9.31E-01	-1.03E-01
pmf0332	Oleanolic acid	1.14E+05	8.09E+04	1.42E+00	7.09E-01	-4.96E-01
pmf0604	β-Caryophyllene	1.38E+07	9.79E+06	1.38E+00	7.11E-01	-4.92E-01
pmf0339	Roseoside	2.15E+06	1.54E+06	1.37E+00	7.15E-01	-4.84E-01
pmf0476	Crocetin	1.34E+04	1.65E+04	1.33E+00	1.24E+00	3.07E-01
pmb2222	Phytocassane C	1.92E+05	2.15E+05	1.04E+00	1.12E+00	1.58E-01
pmf0474	Geniposidic acid	1.03E+05	9.25E+04	9.08E-01	8.99E-01	-1.53E-01
pmf0509	(-)-Camphor	6.54E+03	4.72E+03	7.81E-01	7.22E-01	-4.69E-01
pmf0423	Sweroside	4.74E+05	4.41E+05	7.68E-01	9.31E-01	-1.04E-01
pmf0449	Loganic acid	3.08E+04	2.71E+04	6.50E-01	8.80E-01	-1.85E-01
pmf0435	Loganin	1.28E+04	1.10E+04	5.30E-01	8.61E-01	-2.16E-01
pmf0505	Maslinic acid	1.41E+04	1.29E+04	4.11E-01	9.14E-01	-1.29E-01
pmf0496	Albiflorin	3.14E+04	2.99E+04	4.08E-01	9.54E-01	-6.84E-02
pmf0433	Swertiamarin	4.20E+04	4.83E+04	3.04E-01	1.15E+00	2.02E-01
pme0062	Cucurbitacin D	4.36E+03	4.54E+03	2.01E-01	1.04E+00	5.98E-02
pmf0511	Capsanthin	2.59E+06	2.59E+06	7.81E-02	1.00E+00	3.71E-04
**Vitamins and derivatives**						

pmf0248	delta-Tocopherol	2.68E+03	9.71E+03	1.48E+00	3.62E+00	1.86E+00
pme1709	Nicotinic Acid Methyl Ester (Methyl Nicotinate)	7.86E+04	5.55E+04	1.56E+00	7.06E-01	-5.02E-01
pme2287	Thiamine pyrophosphate	8.41E+03	1.44E+04	1.46E+00	1.71E+00	7.74E-01
pme2167	Orotic acid	1.52E+05	2.59E+05	1.57E+00	1.71E+00	7.73E-01
pme1949	Riboflavin	3.18E+04	2.14E+04	1.34E+00	6.73E-01	-5.71E-01
pme2266	Biotin	3.94E+04	5.40E+04	1.15E+00	1.37E+00	4.56E-01
pmb0802	D-Pantothenic acid	2.80E+06	3.53E+06	1.21E+00	1.26E+00	3.36E-01
pmb0789	Pyridoxine O-glucoside	2.89E+06	3.33E+06	1.30E+00	1.15E+00	2.01E-01
pma1751	Nicotinic acid-hexoside	2.49E+06	2.80E+06	1.15E+00	1.12E+00	1.66E-01
pme3478	Pantetheine	2.38E+05	2.20E+05	9.77E-01	9.24E-01	-1.14E-01
pmb0800	Niacinamide	1.24E+06	1.19E+06	8.81E-01	9.63E-01	-5.44E-02
pma3101	Nicotinate ribonucleoside	4.82E+06	5.73E+06	7.43E-01	1.19E+00	2.51E-01
pme1137	6-hydroxynicotinic acid	1.66E+04	2.06E+04	7.33E-01	1.24E+00	3.06E-01
pme2111	L-ascorbate	1.48E+05	1.37E+05	6.21E-01	9.29E-01	-1.06E-01
pmb0952	Thiamine	1.28E+06	1.19E+06	4.47E-01	9.29E-01	-1.06E-01
pme0490	Nicotinic acid	1.17E+05	1.13E+05	3.73E-01	9.69E-01	-4.59E-02
pmb0801	4-Pyridoxic acid O-hexoside	4.02E+05	4.10E+05	3.43E-01	1.02E+00	2.72E-02
pme2661	Vitamin D3	1.68E+04	1.26E+04	2.84E-01	7.51E-01	-4.14E-01
pme1383	Pyridoxine	5.06E+05	5.04E+05	1.65E-01	9.97E-01	-4.33E-03

Table S3. Differentially accumulated carbohydrates with VIP (variable importance in projection) ≥ 1 as for upregulation/downregulation in the 'Zibao' female flower tissues (CK) by bagging (BF)

Carbohydrates name	Content		Log_2FC (BF vs. CK)	VIP	Type
	CK	BF			
D(-)-Threose	5.60E+03	2.07E+04	1.89E+00	1.03E+00	up
Galactinol	1.24E+06	1.65E+06	4.08E-01	1.18E+00	down
D-Fructose 6-phosphate-disodium salt	5.53E+06	4.61E+06	-2.62E-01	1.17E+00	down
Glucose-1-phosphate	5.63E+06	4.61E+06	-2.89E-01	1.27E+00	down
Ribulose-5-phosphate	2.35E+05	1.86E+05	-3.36E-01	1.45E+00	down
D-Fructose 6-phosphate	4.21E+06	3.22E+06	-3.89E-01	1.40E+00	down
D-Glucose 6-phosphate	7.07E+06	4.93E+06	-5.19E-01	1.60E+00	down

Table S4. Summary of the sequencing assembly

**Sequences**	**CK**	**BF**
**Before trimming**		
Total nucleotides (bp)	7,394,743,914	9,582,970,682
Number of raw reads	48,971,814	63,463,382
Q20 percentage (%)	95.91	97.05
**After trimming**		
Total nucleotides (bp)	3,634,066,244.5	9,137,841,606
Number of raw reads	6,747,389,564	61,519,498
Q20 percentage (%)	98.02	98.43
**Mapping ratio**		
Total nucleotides (nt) of transcripts (bp)	47,519,894	61,519,498
Mapped nucleotides (nt) of transcripts (bp)	43,589,627	56,178,166
Mapped rate (%)	91.37	91.32

Table S5. Significant KEGG pathways (corrected *P*-value ≤ 0.05) of differentially expressed genes (DEGs) in ‘Zibao’ female flower tissues (CK) by bagging (BF)

**No.**	**Pathway**	**DEGs with pathway annotation***	**All genes with pathway annotation**	***P*-value**	**Corrected *P*-value**	**Pathway ID**
1	Plant hormone signal transduction	28	227	1.28E-05	0.000872223	ko04075
2	alpha-Linolenic acid metabolism	14	66	1.18E-05	0.000872223	ko00592
3	Estrogen signaling pathway	17	92	7.17E-06	0.000872223	ko04915
4	Protein processing in endoplasmic reticulum	36	318	4.70E-06	0.000872223	ko04141
5	Plant-pathogen interaction	27	276	0.00059171	0.032307305	ko04626
6	Antigen processing and presentation	15	117	0.00087592	0.039854575	ko04612

*FDR < 0.05 and absolute value of Log_2 ratio ⩾ 2 (2-fold) as the threshold.

Table S6. Expression of genes related to amino acid synthesis and transport Log2FC ≥ 1 and ≤ -1 as for as for upregulation/downregulation in the cv. 'Zibao' CK and BF

Gene ID	FPKM		Log2FC (BF vs. CK)	p-value	Type	Gene Description
	CK	BF				
c44239_g1	0.377	6.895	3.87	2.19E-07	up	Amino acid transporter-like protein 1
c18203_g1	0	0.634	2.88	9.64E-05	up	Amino acid transporter-like protein 1
c45017_g1	4.877	21.797	2.14	1.85E-03	up	Bidirectional amino acid transporter 1
c32134_g2	12.79	3.981	-1.66	4.01E-03	down	IAA-amino acid hydrolase ILR1
c18388_g1	66.91	16.272	-2.03	2.39E-03	down	Jasmonic acid-amido synthetase JAR1
c32501_g1	8.301	1.829	-2.12	1.82E-03	down	IAA-amino acid hydrolase ILR1-like 4
c41192_g1	45.298	5.847	-2.93	1.96E-06	down	Probable vacuolar amino acid transporter YPQ1

Supplementary Figure legends

Figure S1. Projections to latent structures-discriminant analysis (OPLS-DA) results in the 'Zibao' female flower tissues (CK) by bagging (BF).

Figure S2. Preliminary analysis of transcriptomics data. (A) Volcano plots of DEGs between CK and BF. (B) GO classification of unigenes between CK and BF. GO categories: Biological Process, Cellular Component and Molecular Function. (C) KEGG pathway class in the CK vs. BF.

Figure S3. RT-qPCR validation. (A) FPKM values of the 15 candidate genes. (B) RT-qPCR analysis of the expression levels of the 15 candidate genes between CK and BF. (C) The correlation coefficient between FC Seq and FC RT-qPCR.

Figure S1

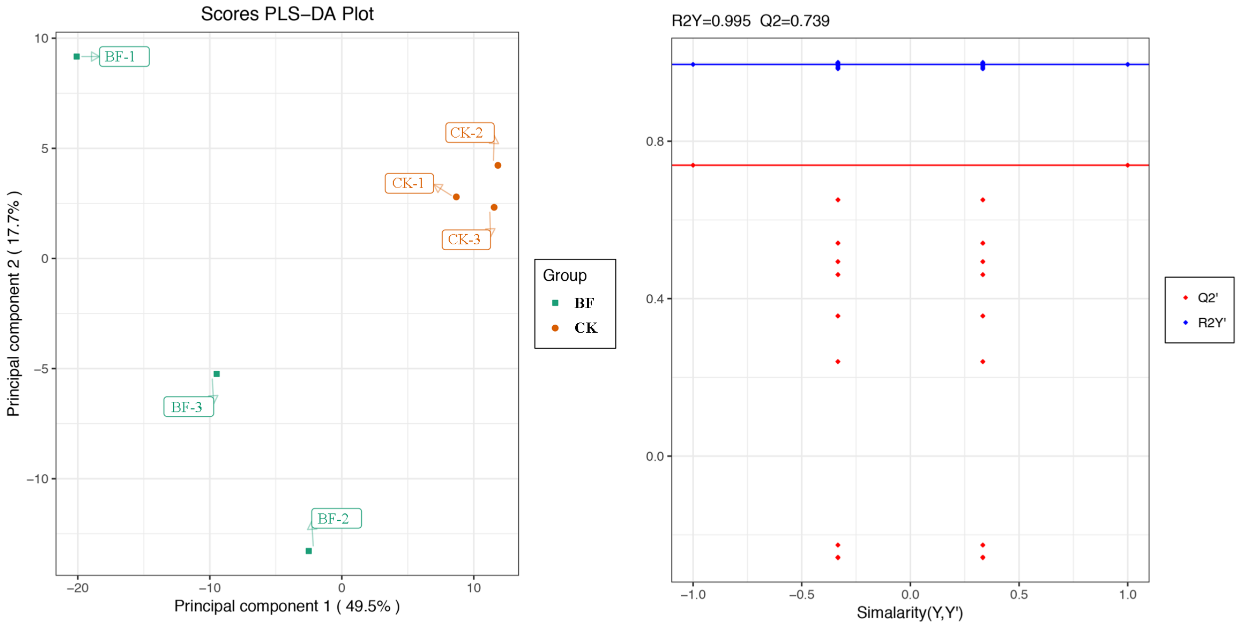
Scores PLS−DA Plot
Principal component 1 (49.5%)
Principal component 2 (17.7%)
BF-1
BF-2
BF-3
CK-1
CK-2
CK-3
Group
BF
CK
R2Y=0.995 Q2=0.739
Simalarity(Y,Y')
Q2'
R2Y'

Figure S2

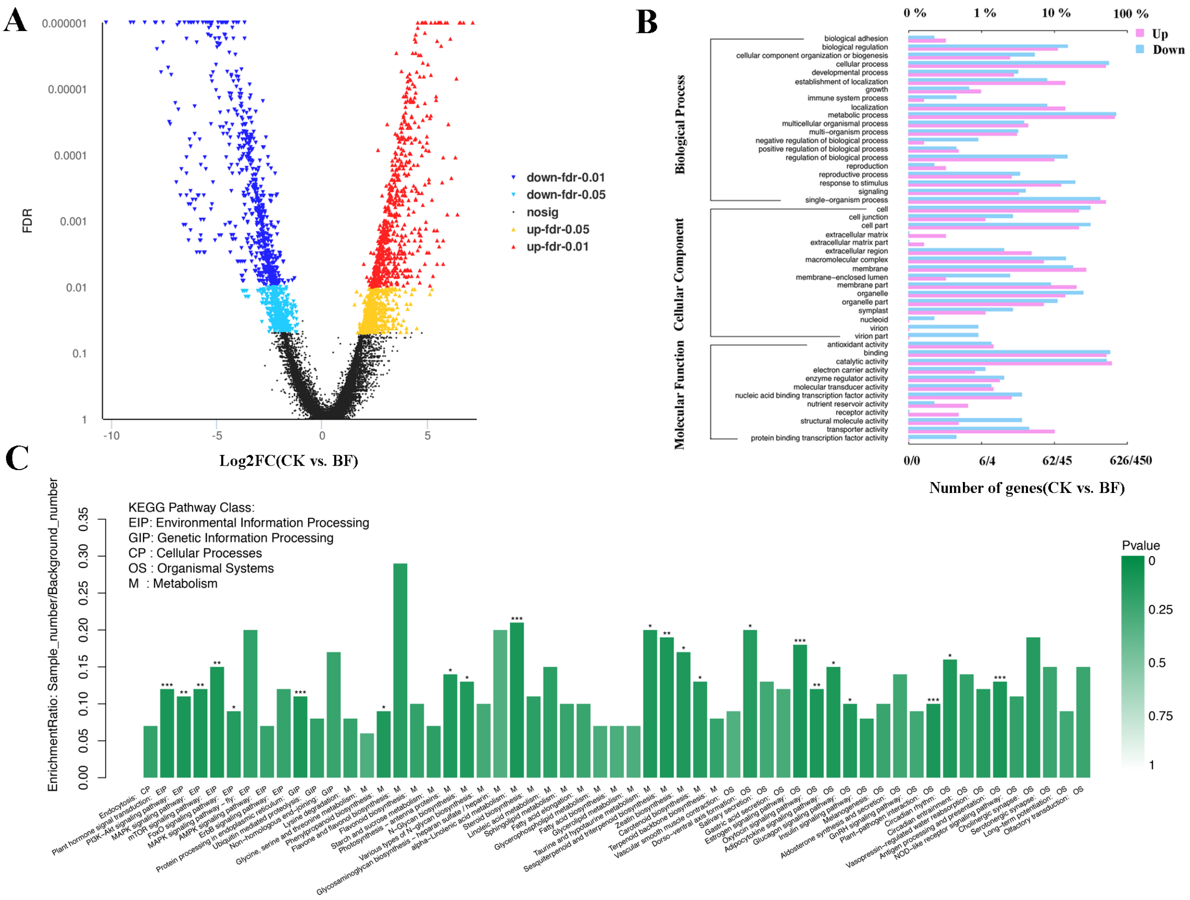

A
B
C
FDR
0.000001
0.00001
0.0001
0.001
0.01
0.1
1
-10
-5
0
5
Log2FC(CK vs. BF)
down-fdr-0.01
down-fdr-0.05
nosig
up-fdr-0.05
up-fdr-0.01
0 %
1 %
10 %
100 %
Up
Down
Biological Process
Cellular Component
Molecular Function
biological adhesion
biological regulation
cellular component organization or biogenesis
cellular process
developmental process
establishment of localization
growth
immune system process
localization
metabolic process
multicellular organismal process
multi–organism process
negative regulation of biological process
positive regulation of biological process
regulation of biological process
reproduction
reproductive process
response to stimulus
signaling
single–organism process
cell
cell junction
cell part
extracellular matrix
extracellular matrix part
extracellular region
macromolecular complex
membrane
membrane–enclosed lumen
membrane part
organelle
organelle part
symplast
nucleoid
virion
virion part
antioxidant activity
binding
catalytic activity
electron carrier activity
enzyme regulator activity
molecular transducer activity
nucleic acid binding transcription factor activity
nutrient reservoir activity
receptor activity
structural molecule activity
transporter activity
protein binding transcription factor activity
0/0
6/4
62/45
626/450
Number of genes(CK vs. BF)
KEGG Pathway Class:
EIP: Environmental Information Processing
GIP: Genetic Information Processing
CP : Cellular Processes
OS : Organismal Systems
M : Metabolism
EnrichmentRatio: Sample_number/Background_number
0.00
0.05
0.10
0.15
0.20
0.25
0.30
0.35
Pvalue
0
0.25
0.5
0.75
1

Figure S3

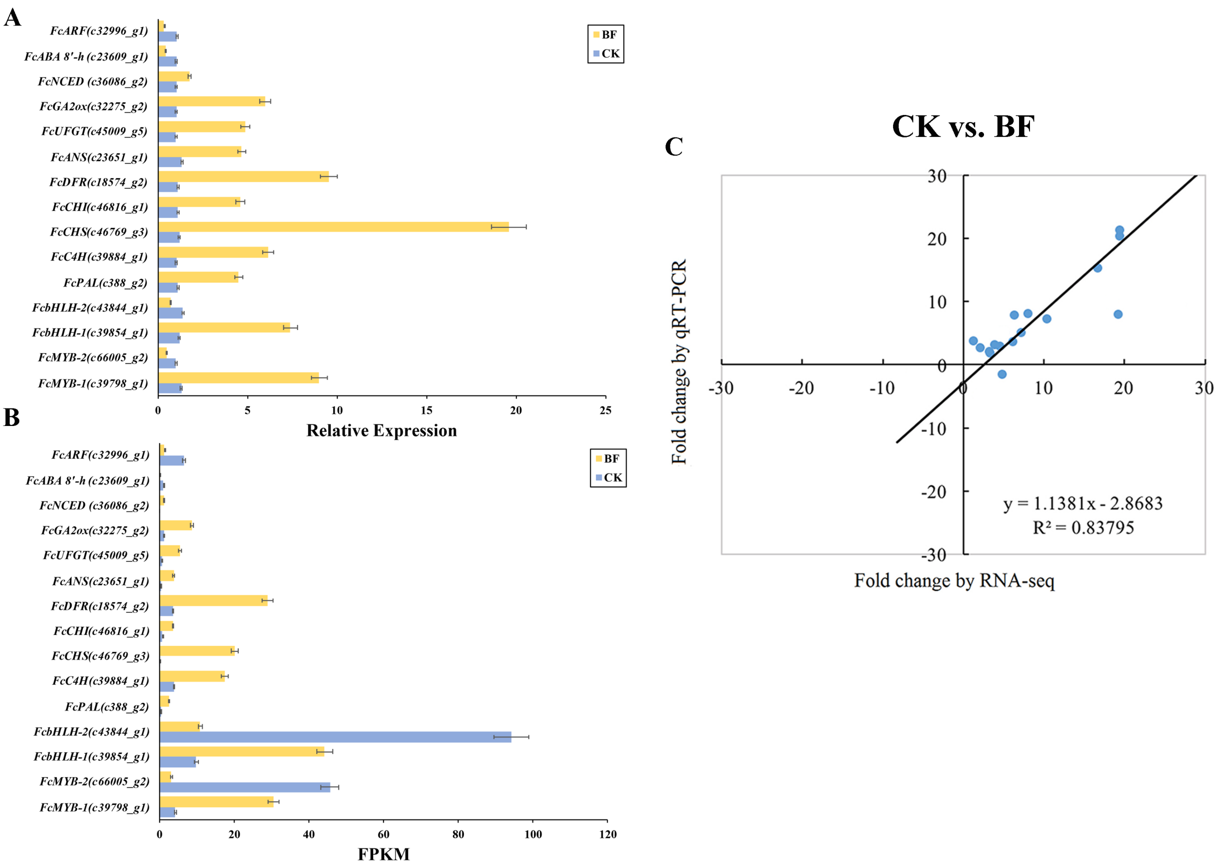
A
FcARF(c32996_g1)
FcABA 8'-h (c23609_g1)
FcNCED (c36086_g2)
FcGA2ox(c32275_g2)
FcUFGT(c45009_g5)
FcANS(c23651_g1)
FcDFR(c18574_g2)
FcCHI(c46816_g1)
FcCHS(c46769_g3)
FcC4H(c39884_g1)
FcPAL(c388_g2)
FcbHLH-2(c43844_g1)
FcbHLH-1(c39854_g1)
FcMYB-2(c66005_g2)
FcMYB-1(c39798_g1)
BF
CK
0 5 10 15 20 25
Relative Expression
B
FPKM
0 20 40 60 80 100 120
C
CK vs. BF
Fold change by qRT-PCR
Fold change by RNA-seq
y = 1.1381x - 2.8683
R² = 0.83795
